# Supplementary material for: Cardiac prehabilitation, rehabilitation and education in first-time atrial fibrillation (AF) ablation (CREED AF): Study protocol for a randomised controlled trial
Source: PLoS One. 2024 Oct 3;19(10):e0310951. doi: 10.1371/journal.pone.0310951 (PMC11449326; doi:10.1371/journal.pone.0310951)
Supplement: S1 Checklist — (DOC) [file pone.0310951.s001.doc]

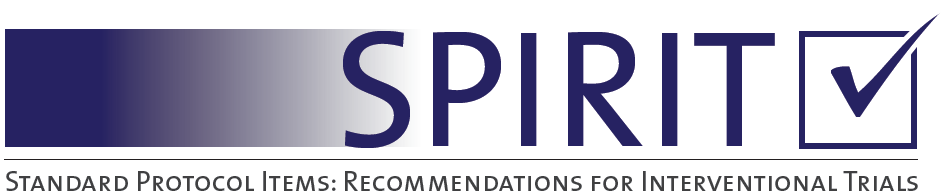


SPIRIT 2013 Checklist: Recommended items to address in a clinical trial protocol and related documents*

| Section/item | ItemNo | Description |
| --- | --- | --- |
| **Administrative information** | | |
| Title | 1 | Cardiac prehabilitation, rehabilitation and education in first-time atrial fibrillation (AF) ablation (CREED AF): study protocol for a randomised controlled trial |
| Trial registration | 2a | https://www.clinicaltrials.gov; Unique identifier: NCT06042231.Date registered: September 18, 2023. |
|  | 2b | All items from the World Health Organization Trial Registration Data Set |
| Protocol version | 3 | CREED AF Protocol v1.1 6.7.23 |
| Funding | 4 | Boston Scientific have provided the funding for this trial. The design and management of this trial are entirely independent of the funder. |
| Roles and responsibilities | 5a | Prof Faizel Osman / Prof Gordon McGregor- Chief Investigators |
|  | 5b | University Hospitals Coventry and Wartwickshire NHS Trust R&D department (UHCW) |
|  | 5c | CI and co-investigators responsible for study design; collection, management, analysis, and interpretation of data; writing of the report; and the decision to submit the report for publication, including whether they will have ultimate authority over any of these activities  Funder has no inflence on the above |
|  | 5d | Composition, roles, and responsibilities of the coordinating centre, steering committee, endpoint adjudication committee, data management team, and other individuals or groups overseeing the trial, if applicable (see Item 21a for data monitoring committee)  As above |
| Introduction |  |  |
| Background and rationale | 6a | Pages 5-6 |
|  | 6b | Pages 6-10 |
| Objectives | 7 | Page 6 |
| Trial design | 8 | Pages 6-7 |
| Methods: Participants, interventions, and outcomes | | |
| Study setting | 9 | Pages 6-7 |
| Eligibility criteria | 10 | Pages 6-7; Figure 3 |
| Interventions | 11a | Pages 9-10 |
|  | 11b | Pages 9-10 |
|  | 11c | Pages 9-10 |
|  | 11d | Pages 9-10 |
| Outcomes | 12 | Pages 11-12 |
| Participant timeline | 13 | Pages 8-10; Figures 1a nd 2 |
| Sample size | 14 | Page 12-13 |
| Recruitment | 15 | Pages 7-8 |
| **Methods: Assignment of interventions (for controlled trials)** | | |
| Allocation: |  |  |
| Sequence generation | 16a | Page 8 |
| Allocation concealment mechanism | 16b | Page 8 |
| Implementation | 16c | Page 8 |
| Blinding (masking) | 17a | Page 8 |
|  | 17b | Page 8 |
| **Methods: Data collection, management, and analysis** | | |
| Data collection methods | 18a | Pages 11-16 |
|  | 18b | Pages 11-16 |
| Data management | 19 | Pages 11-16 |
| Statistical methods | 20a | Pages 12-13 |
|  | 20b | Pages 12-13 |
|  | 20c | Pages 12-13 |
| **Methods: Monitoring** | | |
| Data monitoring | 21a | Pages 18-19 |
|  | 21b | Pages 18-19 |
| Harms | 22 | Pages 18-19 |
| Auditing | 23 | Pages 18-19 |
| Ethics and dissemination | | |
| Research ethics approval | 24 | Page 17 |
| Protocol amendments | 25 | Page 17 |
| Consent or assent | 26a | Page 17 |
|  | 26b |  |
| Confidentiality | 27 | Page 17 |
| Declaration of interests | 28 | Page 18 |
| Access to data | 29 | Page 18 |
| Ancillary and post-trial care | 30 |  |
| Dissemination policy | 31a | Page 18 |
| Appendices |  |  |
| Informed consent materials | 32 | See protocol |
| Biological specimens | 33 | n/a |

*It is strongly recommended that this checklist be read in conjunction with the SPIRIT 2013 Explanation & Elaboration for important clarification on the items. Amendments to the protocol should be tracked and dated. The SPIRIT checklist is copyrighted by the SPIRIT Group under the Creative Commons “[Attribution-NonCommercial-NoDerivs 3.0 Unported](http://www.creativecommons.org/licenses/by-nc-nd/3.0/)” license.
