# Supplementary material for: Cardiac prehabilitation, rehabilitation and education in first-time atrial fibrillation (AF) ablation (CREED AF): Study protocol for a randomised controlled trial
Source: PLoS One. 2024 Oct 3;19(10):e0310951. doi: 10.1371/journal.pone.0310951 (PMC11449326; doi:10.1371/journal.pone.0310951)
Supplement: S1 File — (DOCX) [file pone.0310951.s002.docx]

**CREED AF**

Impact of **C**ardiac prehabilitation, **RE**habilitation and patient **ED**ucation on outcomes in patients undergoing first-time **AF** ablation

**PROTOCOL**

| **IRAS number** | | 318220 | | |
| --- | --- | --- | --- | --- |
| **ClinicalTrials.gov number** | | *<insert>* | | |
| **Sponsor** | | University Hospitals Coventry & Warwickshire NHS Trust | | |
| **Sponsor reference** | | FO596122 | | |
| **Funder** | | Boston Scientific Limited | | |
| **REC reference** | | *<insert>* | | |
| **REC approval date** | | *<insert>* | | |
| **HRA approval date** | | *<insert>* | | |
| **Planned Start date** | | April 2023 | | |
| **Planned End date** | | September 2025 | | |
| **Protocol Amendments** | | | | |
| **Amendment Number** | **Protocol Version** | | **Date of Amendment** | **Date of Approval** |
|  |  | |  |  |

**Confidentiality statement**

All information contained within this document is regarded as, and must be kept, confidential. No part of this document may be disclosed to any Third Party without the written permission of the Chief Investigator and/or Sponsor.

# **SIGNATURE PAGE**

The undersigned confirm that the following protocol has been agreed and accepted and that the Chief Investigator agrees to conduct the trial in compliance with the approved protocol and will adhere to the principles outlined in the UK Policy Framework for Health and Social Care Research, the ICH Good Clinical Practice guidelines, and the Sponsor’s SOPs.

I agree to ensure that the confidential information contained in this document will not be used for any other purpose other than the evaluation or conduct of the clinical investigation without the prior written consent of the Sponsor.

I also confirm that I will make the findings of the study publicly available through publication or other dissemination tools without any unnecessary delay and that an honest accurate and transparent account of the study will be given; and that any discrepancies from the study as planned in this protocol will be explained.

| **For and on behalf of the Study Sponsor:** | | |
| --- | --- | --- |
| Signature:  ........................................................................ |  | Date:  ......../......../...... |
| Name (please print):  ......................................................................... |  |  |
| Position:  ......................................................................... |  |  |
| **Chief Investigator:** | | |
| Signature:  ......................................................................... |  | Date:  ......../......../...... |
| Name: (please print):  .........................................................................  Position:  ........................................................................ |  |  |

# **KEY TRIAL CONTACTS**

| **Chief Investigator** | **Professor Faizel Osman**  University Hospitals Coventry & Warwickshire (UHCW) NHS Trust  Clifford Bridge Road  Coventry  CV2 2DX  [Faizel.osman@uhcw.nhs.uk](mailto:Faizel.osman@uhcw.nhs.uk) |
| --- | --- |
| **Co-investigators** | **Dr Gordon McGregor**  UHCW NHS Trust  [Gordon.McGregor@uhcw.nhs.uk](mailto:Gordon.McGregor@uhcw.nhs.uk)  **Helen Eftekhari**  UHCW NHS Trust  [Helen.eftekhari@uhcw.nhs.uk](mailto:Helen.eftekhari@uhcw.nhs.uk)  **Dr Asad Ali**  UHCW NHS Trust  [Asad.ali@uhcw.nhs.uk](mailto:Asad.ali@uhcw.nhs.uk)  **Joanna Shakespeare**  UHCW NHS Trust  [Joanna.shakespeare@uhcw.nhs.uk](mailto:Joanna.shakespeare@uhcw.nhs.uk)  **Edward Parkes**  UHCW NHS Trust  [Edward.parkes@uhcw.nhs.uk](mailto:Edward.parkes@uhcw.nhs.uk)  **Dr Siew Wan Hee**  UHCW NHS Trust  [SiewWan.Hee@uhcw.nhs.uk](mailto:SiewWan.Hee@uhcw.nhs.uk)  **Dr Angela Noufaily**  University of Warwick  [a.noufaily@warwick.ac.uk](mailto:a.noufaily@warwick.ac.uk)  **Dr Shilpa Patel**  UHCW NHS Trust  [shilpa.patel@uhcw.nhs.uk](mailto:shilpa.patel@uhcw.nhs.uk)  **Dr Lazaros Andronis**  University of Warwick  [l.andronis@warwick.ac.uk](mailto:l.andronis@warwick.ac.uk)  **Dr Nakul Chandan**  UHCW NHS Trust  [Nakul.chandan@uhcw.nhs.uk](mailto:Edward.parkes@uhcw.nhs.uk)  **Dr Hejie He**  UHCW NHS Trust  [Hejie.he@uhcw.nhs.uk](mailto:Hejie.he@uhcw.nhs.uk) |
| **Sponsor** | **Sonia Kandola**  Research and Development Department  UHCW NHS Trust  Clifford Bridge Road  Coventry  CV2 2DX  [ResearchSponsorship@uhcw.nhs.uk](mailto:ResearchSponsorship@uhcw.nhs.uk)  Tel: 02476 966195 |
| **Funder** | **Boston Scientific Limited**  100 New Bridge Street, London, EC4V 6JA  Tel: +44 1442 411 600 |
| **Trial Coordination** | **Trial Management Unit**  UHCW NHS Trust  Clifford Bridge Road  Coventry  CV2 2DX  [creedafstudyinbox@uhcw.nhs.uk](mailto:creedafstudyinbox@uhcw.nhs.uk)  Tel: 02476 966907 |

# **STUDY SUMMARY**

| **Full study title** | Impact of **C**ardiac prehabilitation, **RE**habilitation and patient **ED**ucation on outcomes in patients undergoing first-time **AF** ablation | |
| --- | --- | --- |
| **Short study title** | **CREED AF** | |
| **Study aim** | To compare a tailored comprehensive patient education and lifestyle/Atrial Fibrillation (AF) risk factor modification programme and cardiac pre/rehabilitation (CREED AF Intervention) vs standard of care for patients with AF who are listed for all first-time AF ablation procedure (cryoballoon or radiofrequency ablation) | |
| **Study design** | Single-centre prospective randomised controlled trial. | |
| **Study participants** | Patients with AF listed for first-time AF ablation. | |
| **Study arms** | **Control arm** – AF ablation procedure (standard care) plus 30-minute education with CREED AF practitioner.  **Intervention arm** – AF ablation procedure (standard care) plus CREED AF intervention before and after AF ablation procedure. CREED AF intervention comprised of tailored cardiac education, prehabilitation (2-3 sessions/week for 6-8 weeks) and rehabilitation (2-3 sessions/week for 6-8 weeks). | |
| **Sample size** | 106 (53 in each arm) | |
| **Planned study period** | 28 months (June 2023 to September 2025) | |
| **Planned recruitment start date** | June 2023 | |
| **Planned recruitment end date** | September 2024 | |
| **Planned study end date** | September 2025 | |
|  | **Objectives** | **Outcome Measures** |
| **Primary** | To assess the effectiveness of comprehensive cardiac prehabilitation, rehabilitation and patient education to improve exercise capacity of AF patients undergoing first-time AF ablation, compared with standard care. | VO_2peak_ measured from cardiopulmonary exercise testing (CPET) at baseline and 10 weeks (±2 weeks) post AF ablation between the standard care and intervention arms. |
| **Secondary** | To explore if CREED AF intervention can improve quality of life (QoL) in AF patients undergoing ablation. | 1. EQ-5D-5L questionnaire 2. Generic QoL questionnaire (SF-36 QoL scale) 3. Disease specific questionnaire (Atrial Fibrillation Effect on Quality-of-Life (AFEQT) |
|  | To assess cost effectiveness of the CREED AF intervention compared with standard care. | Incremental cost per quality adjusted life year (QALY). |
|  | To explore if CREED AF intervention has an impact on the short- and long-term recurrence of AF post-ablation and the need for redo-AF ablation. | AF recurrence and burden from data collected from a 1–7-day(s) cardiac Holter monitoring at 10 weeks (±2 weeks) and 12 months (±4 weeks) post ablation, and requirement for redo-AF ablation as documented in medical records reviewed at 12 months post-ablation. |
|  | To explore the impact of CREED AF intervention on relevant major adverse cardiovascular events (MACE) in AF patients. | MACE at 12 months post-ablation, defined as:   1. Hospital admission related to AF 2. Non-fatal myocardial infarction 3. Non-fatal stroke 4. Cardiovascular death 5. All-cause mortality |

Key Words: **AF ablation, Pulmonary vein isolation, VO2peak**

# **STUDY FLOW CHART**

*Figure 1: Flow of participants through the study*


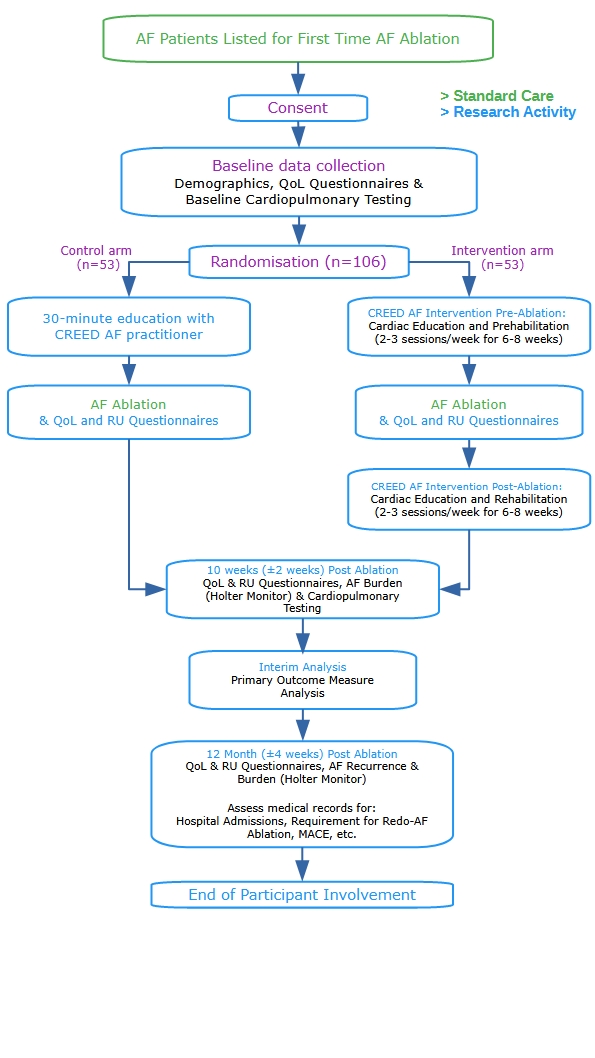


# **SCHEDULE OF EVENTS**

*Table 1: Schedule of Events*

| **Procedure** | **Pre-Screening/ Lead-In Period** | **Consent & Baseline** | **Pre-Ablation Intervention** | **AF Ablation Procedure*** | **Rest** | **Post-Ablation Intervention** | **Follow-Up & Outcome Measure** | **Follow-Up** |
| --- | --- | --- | --- | --- | --- | --- | --- | --- |
|  | **Month -4 to Month -1** | **Week -2 to Day 1** | **Up to Week 8** | **Week 9 (Day 1 Ablation)** | **Up to Week 11 (2 weeks post-ablation)** | **Up to Week 19 (Up to week 10 post-ablation)** | **Up to Week 21 (Up to week 12 post-ablation)** | **Month 12 (±4weeks) post ablation** |
| Screening | X |  |  |  |  |  |  |  |
| Eligibility assessment | X |  |  |  |  |  |  |  |
| Re-Confirm Eligibility |  | X |  |  |  |  |  |  |
| Informed consent |  | X |  |  |  |  |  |  |
| Questionnaire completion (EQ-5D, SF-36, AFEQT) |  | X |  | X |  |  | X | X |
| Resource Use Questionnaire |  |  |  | X |  |  | X | X |
| Demographic data (Year of birth, sex, ethnicity, height, and weight) |  | X |  |  |  |  |  |  |
| Smoking and diet history |  | X |  |  |  |  |  |  |
| Relevant clinical history |  | X |  |  |  |  |  |  |
| Current medications |  | X |  |  |  |  |  |  |
| Standard blood tests* |  | X |  |  |  |  |  |  |
| Cardiopulmonary exercise testing |  | X |  |  |  |  | X |  |
| Randomisation |  | X |  |  |  |  |  |  |
| 1-to-1 behavioural & motivational education sessions |  |  | X^1^ |  |  | X^1^ |  |  |
| 30-minute AF risk factor modification education session |  |  | X^2^ |  |  |  |  |  |
| AF Ablation* |  |  |  | X |  |  |  |  |
| Cardiopulmonary exercise sessions for intervention arm |  |  | X |  |  | X |  |  |
| Adverse Event Reporting |  | X | X | X | X | X | X | X |
| 1–7-day(s) Holter monitor |  |  |  |  |  |  | X | X |
| Primary Outcome Analysis |  |  |  |  |  |  | X |  |
| Review of hospital records (MACE, need for redo-AF ablation, etc.) |  |  |  |  |  |  |  | X |

** Part of Standard Care*

*X^1^ 1-to-1 behavioural & motivational education session alternating weeks for 6-8 weeks pre- and post-ablation only occurring in the intervention arm*

*X^2^ one off 30-minute education session activity only occurring in the control arm*

**CONTENTS**

[**SIGNATURE PAGE** 2](#_Toc131866576)

[**KEY TRIAL CONTACTS** 3](#_Toc131866577)

[**STUDY SUMMARY** 5](#_Toc131866578)

[**STUDY FLOW CHART** 7](#_Toc131866579)

[**SCHEDULE OF EVENTS** 8](#_Toc131866580)

[**LIST OF ABBREVIATIONS** 11](#_Toc131866581)

[**1.** **INTRODUCTION** 12](#_Toc131866582)

[**2.** **BACKGROUND** 13](#_Toc131866583)

[**3.** **RATIONALE** 17](#_Toc131866584)

[**4.** **METHODS** 19](#_Toc131866585)

[**5.** **OBJECTIVES AND OUTCOME MEASURES** 22](#_Toc131866586)

[**6.** **STUDY DESIGN** 24](#_Toc131866587)

[**7.** **STUDY SETTING** 24](#_Toc131866588)

[**8.** **ELIGIBILITY CRITERIA** 24](#_Toc131866589)

[**9.** **TRIAL PROCEDURES** 25](#_Toc131866590)

[**10.** **STATISTICS AND DATA ANALYSIS** 33](#_Toc131866591)

[**11.** **DATA MANAGEMENT** 36](#_Toc131866592)

[**12.** **TRIAL OVERSIGHT** 38](#_Toc131866593)

[**13.** **ADVERSE EVENT MANAGEMENT** 40](#_Toc131866594)

[**14.** **MONITORING, AUDIT & INSPECTION** 44](#_Toc131866595)

[**15.** **ETHICAL AND REGULATORY CONSIDERATIONS** 44](#_Toc131866596)

[**16.** **DISSEMINATION POLICY** 45](#_Toc131866597)

[**17.** **REFERENCES** 46](#_Toc131866598)

# **LIST OF ABBREVIATIONS**

| AE | Adverse Event |
| --- | --- |
| AR | Adverse Reaction |
| AF | Atrial Fibrillation |
| AFEQT | Atrial Fibrillation Effect on Quality of Life |
| BP | Blood Pressure |
| CI | Chief Investigator |
| CPET | Cardiopulmonary Exercise Test |
| CRF | Case Report Form |
| ECG | Electrocardiogram |
| EDC | Electronic Data Capture |
| GCP | Good Clinical Practice |
| ICF | Informed Consent Form |
| ISF | Investigator Site File |
| MACE | Major Adverse Cardiovascular Events |
| MCS | Mental Component Summary |
| NICE | National Institute for Clinical Excellence |
| PCS | Physical Component Summary |
| PI | Principal Investigator |
| PIS | Participant Information Sheet |
| PVI | Pulmonary Vein Isolation |
| QALY | Quality Adjusted Life Year |
| QoL | Quality of Life |
| RCT | Randomised Control Trial |
| REC | Research Ethics Committee |
| RU | Resource Use |
| SAE | Serious Adverse Event |
| SAR | Serious Adverse Reaction |
| SDV | Source Data Verification |
| SOP | Standard Operating Procedure |
| SUSAR | Suspected Unexpected Serious Adverse Reaction |
| TMF | Trial Master File |
| TMG | Trial Management Group |
| UHCW | University Hospitals Coventry & Warwickshire |
| VAS | Visual Analogue Scale |

**STUDY PROTOCOL**

Impact of **C**ardiac prehabilitation, **RE**habilitation and patient **ED**ucation on outcomes in patients undergoing first-time **AF** ablation

# **INTRODUCTION**

In the UK, the National Institute of Clinical Excellence (NICE) have provided guidance for atrial fibrillation (AF) management, including AF ablation. First-line treatment for AF is pharmacological using rate-controlling and/or anti-arrhythmic medications. Whilst medications can be used successfully, they are not always successful, suitable, or tolerated, and ablation procedures can be considered. However, an AF ablation is not without risk and carries a success rate of 50-80% for a first-time procedure with an attrition rate over time with a recurrence of AF seen at longer term follow-up (1). Additionally, AF ablation also has significant cost implications for healthcare providers worldwide. These can often be offset by performing day-case AF ablation. We have reported the safety and efficacy of day-case standard ablation at our centre (2) and as well as performing day-case AF ablation (3).

A recent NICE Clinical Commissioning Policy Proposition for catheter ablation of atrial fibrillation explored the evidence for catheter and surgical AF ablation vs medical therapy and aims to provide recommendations on the suitability of patients for such treatments (4). The review is evaluating whether ablation of AF is justified in terms of cost effectiveness. The success rate of catheter ablation for persistent AF at 1-year is 40-60% and 70-80% for paroxysmal AF; this and the known attrition over time are factors that significantly impact the ability of healthcare providers (such as the NHS) to be willing to pay for these expensive treatments. There is concern that this may restrict who and how many patients can undergo an AF ablation procedure in the future.

Our proposal aims to establish whether a simple and relatively inexpensive non-invasive intervention (AF risk factor modification using patient education and cardiac pre/rehabilitation - termed ‘CREED AF intervention’) targeted at AF patients before and after a first-time planned AF ablation can improve short- and medium-term physical and psychological outcome measures (5). If outcomes are improved following the proposed ‘CREED AF intervention’ there may be significant benefits for both patient and healthcare providers in relation to higher short- and medium-term success rates of AF ablation. This in turn could lead to a stronger recommendation for AF ablation as first-line therapy in these patients and lead to changes in national and international guidelines on recommendations for such treatments, which could be carried out in a wider group of patients worldwide.

# **BACKGROUND**

Atrial Fibrillation

Atrial fibrillation is one of the most common heart rhythm disturbances (arrhythmias) worldwide (6–8); currently affecting nearly 3% of the western population. AF rates are predicted to increase over the coming decades with an increase from 7 million to almost 13 million affected people by 2030 (9–11). AF is independently associated with increased morbidity and all-cause mortality with concomitant ischaemic stroke, dementia, cognitive dysfunction, heart failure and ischaemic heart disease (8–13). Stroke and heart failure can be the first manifestation of AF (12,14). The growing AF epidemic is due to the ageing population, the accumulation of AF related risk factors (for example diabetes, obesity, and high blood pressure) and chronic cardiovascular diseases (11,15,16). The NHS 2019 long term plan recognises the long-term healthcare implications of the increasing AF burden, and the importance of specifically targeted management of AF.

Atrial Fibrillation can present heterogeneously and can be split into five diagnostic types (8):

1. First-diagnosed AF: patients presenting with AF for the first time.
2. Paroxysmal AF (PAF): AF that is self-limiting and usually reverts. spontaneously to sinus (normal) rhythm within 48 hours but can last up to 7 days.
3. Persistent AF: AF lasts longer than 7 days, or requires cardioversion with drugs or direct current cardioversion.
4. Long-standing persistent AF: duration of AF is >1 year and a rhythm control strategy is adopted.
5. Permanent AF: AF that is accepted by the patient (and physician) and no rhythm control interventions are used. Should a rhythm control strategy be adopted, the arrhythmia is reclassified as ‘long-standing persistent AF’.

Whilst patients can present with persistent AF at the time of diagnosis, AF is believed to be a progressive disease developing from a paroxysmal, self-terminating form through persistent to permanent AF perpetuated by on-going electrical and structural remodelling of the atria (17–19).

AF can be completely asymptomatic, however, nearly two-thirds of patients experience at least intermittent symptoms which can be disabling and markedly impair health-related quality of life (14,20). Symptoms include palpitation, shortness of breath, fatigue, dizziness and syncope (fainting), depression, anxiety, and reduced exercise capacity. AF related symptoms and complications, as well as underlying cardiovascular conditions lead to unplanned hospital admissions in a substantial number of patients every year and accounts for more than two-thirds of annual direct costs and is a major cost driver in the NHS (21,22).

AF Management

Currently the management and treatments for AF has focused on preventing AF recurrence and the associated consequences. This includes stroke and heart failure prevention, ventricular rate control, and rhythm control therapies through: anti-arrhythmic drugs, and catheter or surgical ablation (23–25). Catheter ablation is becoming increasingly more common as an AF treatment option. Ablation is invasive, involving either freezing or burning microscopic tissue in the heart responsible for the initiation of AF, with a success rate for AF of about 40-80% (23).

Whilst this approach has proved its efficacy it often fails to address key patient and health outcome measures such as exercise capacity, health-related quality of life (HRQoL), lifestyle indices, ability to self-manage, as well as mental health. It has been argued that AF management should no longer solely address single domains such as stroke prevention, symptom relief or preservation of left ventricular function, but instead target a broader integrated approach which incorporates timely detection and optimal treatment of risk factors and underlying conditions to prevent progression of disease and reduce AF burden in a more fundamental way. This is reflected in the current European Society of Cardiology AF guideline which outlines five domains for AF management and treatment (20). These include acute rhythm management in patients presenting with hemodynamic instability, detection and treatment of underlying predisposing conditions, stroke risk assessment, and oral anticoagulation for stroke prevention, rate control and rhythm control. The second domain now puts management of risk factors and underlying conditions in a much broader prospective (8).

Recent studies have identified risk factor modification (such as hypertension, diabetes, smoking, alcohol intake, weight, and obstructive sleep apnoea) and patient education to be crucial components in managing AF (1-4). Nurse-led education and risk factor modification programmes have shown to play a crucial role in reducing AF burden, hospitalisations, improving medication adherence and quality of life (26–28). Furthermore, the outcome of catheter ablation has been directly linked to the presence of risk factors (or co-morbidities) including those listed above. Prospective studies have also demonstrated active management of these risk factors leads to the improvement of arrhythmia-free survival after catheter ablation (29).

However, at present AF patients do not receive cardiac rehabilitation as part of standard care. The proposed ‘CREED AF intervention’ may improve key patient and health related outcomes (quality of life, prevent AF recurrence, physical activity etc.) and may also drive down the direct costs for AF management, and benefit healthcare services worldwide.

Cardiac rehabilitation is currently a recognised and integral part of comprehensive care for ischaemic heart disease patients and has been given a Class 1 recommendation from the American Heart Association, the American College of Cardiology, and the European Society of Cardiology, with exercise and an education programme consistently identified as central elements (27,28,30,31). The NHS long term plan also prioritises cardiac rehabilitation for patient groups other than the traditional ischaemic heart disease patient. Exercise training is a cornerstone intervention, and comprehensive rehabilitation includes education, risk factor analysis and modification, and psychological input. A recent Cochrane systematic review investigating the effectiveness of cardiac rehabilitation in patients with ischaemic heart disease (32) reported that it provided important health benefits that include reduction in cardiovascular mortality, hospitalisation and associated healthcare costs whilst improving health related quality of life. In recent years, the studies investigating the impact of exercise-based cardiac rehabilitation in AF patients has risen but, there is no consolidation of findings. While authors have claimed positive results, it is hard to draw definitive conclusions as to the effectiveness of cardiac rehabilitation regarding key patient and health outcomes due to the following:

1. The studies have used a wide variety of exercise-based interventions (i.e., aerobic, high intensity exercise, high intensity exercise vs low intensity exercise, yoga & Qi gong).
2. Studies have included a diverse population of patients having different types of AF (paroxysmal, persistent, or permanent) making it difficult to consolidate the findings of these studies.
3. Studies do not have sufficient participants to evaluate the key patient and health outcome measures such as cardiac mortality or reduction in hospitalisation.
4. There has been a lack of focus on education, risk factor modification and psychological support.
5. There has been a lack of patient and public involvement in intervention development.

In a recent Cochrane systematic review and meta-analysis investigating randomised control trials exploring cardiovascular rehabilitation in AF, authors commented there was insufficient evidence to determine the effectiveness of cardiac rehabilitation in AF (33). Currently there is not enough evidence to assess the impact of exercise on the risk of mortality or hospitalisation, or to determine the optimal exercise training intensity to elicit beneficial health outcomes (34,35).

Studies have attempted to explore the efficacy of nurse-led AF education programmes in risk factor management and healthcare outcomes. Authors have reported improvement in quality of life, reduction in hospital visit, improved medication adherence and reduced mortality. In a recent systematic review exploring the efficacy of nurse led education programme in AF reported improvement in key patient and health care outcomes (26–28). At present there is a lack of studies exploring the efficacy of education and multiple risk factor modification in combination with cardiac rehabilitation prior to AF ablation.

Prehabilitation

Efforts to improve outcomes have traditionally been focused on the postoperative period (rehabilitation). However, in recent years studies have suggested that this may not be the most opportune time to commence lifestyle changes as patients may be fatigued, concerned about disturbing the healing process of their intervention, and anxious as they await additional treatments for underlying conditions. The pre-operative period may in fact be a pertinent time to intervene, as patients may be in a better condition physically as compared with post-operatively. In some cases, they may even have a prolonged waiting period for their surgery (36,37). The process of enhancing an individual functional capacity before scheduled surgery, aimed at improving patients’ tolerance to upcoming physiological stress, has been coined ‘prehabilitation’. At present we lack the evidence for the efficacy of prehabilitation in AF patients undergoing AF ablation. However, there is evidence to show the benefits of prehabilitation (or prehab) in patients undergoing surgeries such as colorectal abdominal surgery and cancer treatments (38–40). The suggested outcome benefits include reduced length of stay, less postoperative pain, and fewer postoperative complications, but the evidence is limited (40). An observational study suggested that compared with a historical control, a 4-week preoperative trimodal intervention comprising moderate-intensity aerobic and resistance exercise, diet counselling with whey protein supplementation, and anxiety-reduction strategies was effective in improving preoperative functional walking capacity and accelerating postoperative recovery. In a recent systematic review exploring the efficacy of prehabilitation prior to major non-cardiac surgery authors have reported that ‘prehabilitation can reduce overall and pulmonary morbidity following surgery and could be utilised routinely’ (41). Our proposed ‘CREED AF intervention’ includes prehabilitation and is the first trial to explore its efficacy in AF patients undergoing initial AF ablation. Our local data show that AF patients at UHCW NHS Trust have a prolonged waiting period to receive their planned AF ablation. During this period patients are not provided with any support for risk factor management or education except pharmaceutical agents.

To summarise, key patient and health outcomes in AF may be improved through a comprehensive exercise-based rehabilitation programme to address the management of risk factors associated with the progression of AF. Additionally, large, randomized trials are needed to enable us to explore the impact on other outcomes, such as mortality, cerebrovascular events, and cardiac hospitalisations. At present there is a lack of such an intervention. Our study attempts to investigate the efficacy of an exercise-based rehabilitation programme with patient education for AF patients undergoing first-time AF ablation.

# **RATIONALE**

- 1. **Hypothesis**

It is hypothesised that the CREED AF intervention will improve key patient and health outcome measures, AF burden and physical activity in AF patients undergoing a first-time AF ablation only as their initial procedure, in line with current guidelines, compared to usual care.

- 1. **Justification**

Over the last few years our understanding of AF aetiologies and mechanisms has increased with the role of modifiable risk factors playing a significant role in AF progression and recurrence being increasingly recognised (31,42). While certain risk factors and predisposing conditions cannot be modified (such as age, ethnicity, gender, or genetic predisposition), several risk factors can be modified and optimally managed. These include: obesity, smoking, excess alcohol consumption, hypertension, diabetes, physical inactivity, and obstructive sleep apnoea. Many of these risk factors are like risk factors for other cardiovascular diseases such as ischaemic heart diseases, vascular disease, and heart failure. Patients suffering from these conditions are offered targeted integrated therapies which incorporate risk factor adjustment and management of lifestyle indices such as cardiovascular rehabilitation, as routine. However, current guidelines for patients undergoing AF ablation do not mandate referral for integrated therapies to provide management for these modifiable AF risk factors. Targeting these modifiable risk factors as early as possible in AF patients who have failed medical therapy could potentially reduce AF burden, reverse atrial remodelling and limit AF progression following an AF ablation procedure. It may also improve underlying co-morbidity and in turn reduce stroke and other cardiovascular adverse events.

As patients with AF have differing modifiable risk factor profiles, with many having more than one modifiable risk factor, non-pharmacological interventions aimed at AF risk factor management (including lifestyle modification and education, treatment/targeting underlying conditions) needs to be patient-centred and tailored to individual needs. Thus, targeted therapy of risk factors and underlying conditions is now being considered an important pillar for the management of AF (31,43–45), especially in patients undergoing invasive procedures such as an AF ablation. The latter carries a small but significant risk from the intervention itself (17-19). The following section discusses the role of these common cardiovascular risk factors and the influence they have on developing AF.

Obesity and AF

The evidence linking obesity as an independent risk factor for AF has grown in recent years. Results from the ARIC study (46) have shown that overweight and obesity (BMI ≥25 kg/m2) accounted for about 18% of AF incidents, making obesity the second strongest AF risk factor. Comparable results have also been reported in other large studies including the WHI observational study and Framingham heart study (47,48). A recent meta-analysis found that there is an increase in risk of 29% for every 5 additional BMI units (49). In addition, there is also a 10% increase in recurrence of postoperative AF as well as a 13% increase in post-ablation AF (49).

Smoking and AF

Numerous cohort studies have tried to explore the link between smoking and the increased risk of AF; however, reported results have varied. Results reported from the ARIC study showed that current smoking accounted for about a 10% increase in the incident of AF (50), whereas other authors have documented an increase in risk of up to 32% in current smokers, with some even doubling this figure (13,43,51). At present conclusive evidence regarding smoking and AF prevention is lacking, however, smoking cessation in general is strongly recommended.

Alcohol Consumption and AF

The causal relationship between alcohol consumption and AF has been known for quite some time, especially acute heavy drinking (52). Furthermore, results of two meta-analyses show a linear dose-response relationship between alcohol intake and incidence of AF (53,54). There was a significant 8% increase in the relative risk of the incident of AF for each standard drink per day, in comparison to no drinks at all (55). A recent study also found that abstinence from alcohol significantly reduced arrhythmia recurrences at 6-month follow-up in regular drinkers with AF (both paroxysmal and persistent) who were in sinus rhythm at baseline (56).

Obstructive Sleep Apnoea and AF

In recent years, obstructive sleep apnoea (OSA) has emerged as one of the novel risk factors for AF (31). Sleep-disordered breathing is a common condition with mild OSA affecting one in five adults and moderate or severe OSA affecting one in 15 (57). It should also be noted that hypertension, diabetes, obesity and advancing age, are common influencing factors for developing both OSA and AF (58). In patients suffering with AF, the prevalence of developing OSA is estimated at about 50% or even higher (59,60). Patients suffering from OSA have a significantly higher risk of developing AF, especially if they have severe disease. A study in patients with OSA and symptomatic AF undergoing AF ablation showed that arrhythmia-free survival was better in those receiving continuous positive air pressure treatment compared with those who are not (61).

# **METHODS**

- 1. **Proposed study**

We aim to explore the effectiveness of a tailored comprehensive cardiac rehabilitation programme (both before and after the planned AF ablation) comprising of an exercise programme and patient education programme incorporating lifestyle behaviour change (CREED AF intervention). The education programme will centre on risk factor analysis with individualised risk factor targeting. This will include weight reduction support, advice on alcohol consumption and smoking cessation support. There will be risk factor modification to optimise hypertension/diabetes control, assess for presence of obstructive sleep apnoea with onward referral if needed, and advice on regular exercise. The education sessions will integrate a psychosocial well-being component to support self-management.

- 1. **Study population**

Patients with AF who are referred for first-time AF ablation at University Hospitals Coventry & Warwickshire NHS Trust.

- 1. **Intervention**

The CREED AF intervention will involve a cardiac pre/rehabilitation exercise training programme combined with a patient education programme.

To ensure generalisability for future trials, provisions will be based on current delivery models of cardiac rehabilitation in cardiovascular diseases. A 6-8-week supported programme of 2-3 times weekly exercise will be delivered with an initial individual assessment for each patient randomised to the intervention. Participants will attend a one-to-one appointment with a ‘CREED AF practitioner’ (specialist cardio-pulmonary clinical exercise physiologist or physiotherapist) for the assessment of medical history, medication, clinical parameters, physical activity history and discussion of participant goals, expectations, fears, and concerns. Patients randomised to the CREED AF intervention will have access to both existing cardio-pulmonary rehabilitation programmes at the Atrium Health Centre, Coventry (i.e., they will exercise alongside people with a range of cardio-pulmonary disorders at already running sessions) AND instructor-led, at home workout sessions that will take place live over a video conferencing software. It is anticipated that participants randomised to the CREED AF intervention will choose to receive a hybrid of the two approaches to reduce travel constraints of getting to and from the Atrium Health Centre, and to work more flexibly around a participant’s working/personal life. To maximise accessibility and resource, whilst ensuring benefits are retained, the CREED AF intervention will be delivered as a ‘rolling’ programme. Participants randomised to the CREED AF intervention can immediately join existing cardio-pulmonary rehabilitation exercise programmes rather than waiting for the recruitment of sufficient numbers to form a discrete group of trial participants.

Cardiac pre/rehabilitation

This will be a tailored, individualised exercise programme within pre-specified parameters. Clinical information, data from the exercise assessment, and patient-centred goal setting will be used to devise a safe and effective exercise prescription which can be performed either using pre-existing cardiopulmonary exercise groups at Atrium Health or using the instructor-led online classes that will be available. Exercise guidance, specific to AF, will be delivered on an individual basis during a 1-to-1 familiarisation exercise session in the first week of the programme, and reinforced throughout, by clinical staff. Familiarisation sessions, conducted within the cardiopulmonary rehabilitation programmes will enable participants to build their confidence and there will be optimisation of the exercise prescribed.

The cardiac rehabilitation can be undertaken within existing cardio-pulmonary rehabilitation programmes delivered by NHS clinical staff. Up to 3 times weekly, one hour, exercise sessions for 6-8 weeks before and for 6-8 weeks after AF ablation, with a quantifiable and progressive dose of individualised, multi-modal, aerobic, muscular strength, endurance and ‘functional fitness’ exercise. Adequate warm-up and cool-down will be incorporated. Intensity can be monitored and adjusted using heart rate (where appropriate), rating of perceived exertion, and dyspnoea scale. There is normally a 1–2-week recovery period after an AF ablation procedure to allow for femoral access site wound healing. Only after the participant has completed this 2-week recovery period will they restart their exercise intervention.

The exercise component will be optimised to be appropriate for a broad spectrum of patients including frailer, deconditioned, low-mobility, exercise-naive participants. Conventional gym exercise will be combined with ‘functional fitness’ training. The programme will be highly adaptable to allow personalisation to lower or higher ability participants, whilst ensuring safety and efficacy. This is widespread practice within cardio-pulmonary rehabilitation programmes in the UK.

In addition to treadmills, cycles, and rowing ergometers, we will make use of readily available functional fitness equipment such as steps, floor agility ladders, low rise balance beams, power bags, plyometric boxes, balls (throw/bounce) etc. We will also utilise a range of body weight exercises for online sessions at home. Central to our intervention is the expertise and experience of the specialist CREED AF practitioner who will ensure holistic, safe, individualised, and effective exercise training.

Education, behavioural and lifestyle AF risk Factor Modification Support

The education programme will be delivered in 1-to-1 sessions with a delegated and qualified member of the research team which can be delivered either face-to-face or via an online video conferencing software. The education session will be delivered as part of the CREED AF intervention. Changing behaviours can be difficult and we recognise the importance of psychological support in AF patients. We will, therefore, incorporate discussions with individuals on motivation to change, barriers to change, goal setting and problem-solving to build self-efficacy and encourage behaviour change, adherence, and compliance to risk factor modification. Every second week (a total of 6 1-to-1 education sessions, 3 sessions in prehabilitation before ablation, and 3 sessions in rehabilitation after ablation), before or after exercise, participants will receive a 1-to-1 30-minute behavioural and motivational session with the aim of improving short- and long-term adherence to exercise and lifestyle modification goals. The aim of this will be to provide help to patients to manage lifestyle risk factors. These will include reduction of alcohol consumption to less than 2 Units per day (in line with current UK government guidelines), ensure good blood pressure control in line with international guidelines (<140/85mmHg), provide smoking cessation support, help with good diabetes control, advice on weight reduction management (through diet and exercise) and identification and treatment of obstructive sleep apnoea (OSA). Patients in the intervention group will be provided with written materials, including but not limited to a patient workbook and publicly available patient information leaflets, with sign-posting to support groups as applicable to each individual.

# **OBJECTIVES AND OUTCOME MEASURES**

| **Objectives** | **Outcome Measures** |
| --- | --- |
| ***Primary*** | |
| To assess if the CREED AF intervention improves exercise capacity (assessed by VO_2peak_) in AF patients listed for first-time AF ablation compared to standard care at 10 weeks (±2 weeks) post-ablation. | The VO_2peak_ is measured as described in Section 9.9 Cardiopulmonary Exercise Testing (CPET) at baseline and 10 weeks (±2 weeks) post-ablation which in the intervention arm coincides with two weeks after the last CREED AF intervention exercise session. |
| ***Secondary*** | |
| To explore if the CREED AF intervention improves short- and long-term EQ-5D-5L in AF patients listed for first-time AF ablation compared to standard care. | The EQ-5D-5L is a health status instrument made up of five dimensions: mobility, self-care, usual activities, pain/discomfort, and anxiety/depression, and a visual analogue scale (VAS). There are 5 response levels within each dimension.  The EQ-5D index value is computed according to the scoring algorithm from EuroQol. The value ranges from -0.148 (worst) to 0.949 (best).  Participants indicate their overall health on the day of questionnaire completion on a vertical EQ VAS which ranges from 0 (the worst health you can imagine) to 100 (‘the best health you can imagine’).  Short-term: change of EQ-5D index value and EQ VAS from baseline to 10 weeks post-ablation.  Long-term: change of EQ-5D index value and EQ VAS from baseline to 12 months post-ablation. |
| To explore if the CREED AF intervention improves short- and long-term 36-item short form health survey (SF-36) in AF patients listed for first-time AF ablation compared to standard care. | SF-36 is made up of eight domains (physical functioning, role-physical, bodily pain, general health, vitality, social functioning, role-emotional, and mental health). Each of the domain contributes to the Physical Component Summary (PCS) and Mental Component Summary (MCS) which ranges from 0 (worst) to 100 (best).  Short-term: change of PCS and MCS and each domain from baseline to 10 weeks post-ablation.  Long-term: change of PCS and MCS and each domain from baseline to 12 months post-ablation. |
| To explore if the CREED AF intervention improves short- and long-term Atrial Fibrillation Effect on Quality-of-Life (AFEQT) in AF patients listed for first-time AF ablation compared to standard care. | The AFEQT is an AF-specific health-related QOL made up of 3 subscales (symptoms, daily activities, and treatment concern). The transformed overall score ranges from 0 (complete disability) to 100 (no disability).  Short-term: change of AFEQT from baseline to 10 weeks post-ablation.  Long-term: change of AFEQT from baseline to 12 months post-ablation. |
| To explore the cost-effectiveness of the CREED AF intervention compared to standard care. | The incremental cost per quality adjusted life year (QALY) will be estimated. See details in Section 10.7. |
| To explore if the CREED AF intervention can decrease AF burden and recurrences following AF ablation, and reduce the requirement for redo-AF ablation. This will be assessed with a 1–7-day cardiac Holter monitor at 10 weeks (±2 weeks) and 12-months (±4 weeks) post ablation, and on review of patients’ medical records at 12-months. | Participants will be fitted with a Holter monitor at the 10-week and 12-month post-ablation follow-ups. Of the list of summary data over the time period it is worn by the participant, only the significant AF event and burden, and when it occurred will be recorded. |
| To explore if the CREED AF intervention has an impact on major adverse cardiovascular events (MACE) at the 12-month follow-up between both groups. MACE will be defined as individual outcomes and as a composite of hospital admission related to AF, non-fatal myocardial infarction, non-fatal stroke, cardiovascular death, and all-cause mortality. | Any occurrence of the following events:   - Hospital admission related to AF (from hospital records) - Non-fatal myocardial infarction (as above) - Any non-fatal stroke (as above) - Cardiovascular death (as above) - All-cause mortality (as above)   and when each occurred. |

# **STUDY DESIGN**

This is a single centre, prospective, randomised controlled trial.

# **STUDY SETTING**

The CREED AF study will run from University Hospitals Coventry & Warwickshire (UHCW) NHS Trust. There will be collaboration with Atrium Health, a non-profit exercise rehabilitation centre located in Coventry, UK. Atrium Health was founded by frontline NHS clinical staff to provide people with tailored exercise and education programmes.

# **ELIGIBILITY CRITERIA**

Eligible patients will be those suffering from AF (including long-standing AF) who are listed for a first-time AF ablation. Building upon existing evidence and input from our PPI, it was felt that due to the heterogeneity of AF presentations and our trial sample size being small it was preferable that the study population was homogenous i.e., only those with AF undergoing a first-time AF ablation. Although medical therapy remains the foundation of the treatment of AF, catheter ablation using Pulmonary Vein Isolation (PVI) is assuming an increasingly greater role (18). The success rate of a first-time AF ablation procedure for AF is estimated at ~40-80% at 1-2 years, after which there is attrition (recurrence of AF) over time; studies have associated this attrition to cardiac risk factors.

- 1. **Inclusion criteria**
- All patients AF listed for a first-time AF ablation procedure
- Patients aged ≥18 years
- Able to understand basic spoken and written English themselves, or with support from family and friends.
  1. **Exclusion criteria**
- Pregnancy
- Lack of capacity to consent and participate
- Presence of contraindications or limiting physical or mental health co-morbidity preventing travel, safe exercise, or productive engagement with the trial
- Unable to access online exercise and support sessions from home
- Participating in a research trial that will impact on their standard AF ablation procedure

# **TRIAL PROCEDURES**

- 1. **Screening**

University Hospitals Coventry and Warwickshire performs ~ 300 ablations per year. Delegated members of the research team, who are also members of the direct care team, will screen the list of participants who are referred to UHCW for an ablation procedure. If the participant meets the inclusion/exclusion criteria then the research team will provide the potential participant with the participant information sheet (PIS) and invitation letter, inviting them to take part in the study. Where applicable, an expression of interest form and consent form will also be provided. Potential participants can be approached either face-to-face during a standard care clinic appointment, or remotely using a method acceptable to the patient e.g., phone, post, email, video call. Reasonable effort will be made to contact the participant following provision of the PIS and invitation letter. Screening logs will be kept by the study team to provide information on patient eligibility, and reasons for non-participation, to inform future NHS service design. Patient’s sex and ethnicity will also be logged at screening to monitor inclusion.

- 1. **Recruitment**

Prospective participants who are eligible to participate will be contacted by a member of the research team to confirm if they are interested in taking part. The member of the research team will explain the study in more detail and answer any questions the prospective participant may have. During the initial consultation, the potential participant will have explained to them the full study procedures should they decide to join the trial. It will be made clear that they could be randomised to the control arm whereby they would not receive the exercise intervention, and only be required to complete the outcome assessments and one 30-minute risk management education session. The prospective participant will be given a suitable amount of time to consider joining the study and encouraged to ask questions. Following the initial consultation, the prospective participant will be invited to join the study. If they agree to take part, they can then be consented. If a participant is consenting over the phone, they will be asked to counter sign their consent form on their first study visit to the hospital.

- 1. **Payment**

Participants will be reimbursed their travel costs for travelling to and from the Atrium Health Centre, for the purpose of CREED AF exercise training visits. They will be reimbursed up to a maximum of £10 per visit to the Atrium Health Centre, which will cover the cost of travel (public transport or fuel) and parking. This can be claimed by the participant at the conclusion of each intervention period (e.g., end of prehabilitation and rehabilitation). We will aim to align hospital visits with standard of care, however if any additional visits to the hospital are required that are specifically for the purpose of the CREED AF study, then these travel visits will be reimbursed, after each visit. Participants will be required to keep a log of their travel expenses during their time in the study.

- 1. **Consent**

Prospective participants will be asked to confirm whether they have read and understood the PIS and will be encouraged to ask questions and be provided enough opportunities to discuss before deciding to take part in the study. Patients will be free to discuss the study with friends and family before reaching their decision.

After confirming the patient’s eligibility to take part, a qualified member of the research team, who has received training in obtaining informed consent, will obtain consent. Informed consent will be obtained prior to the participant undergoing procedures that are specifically for the purposes of the CREED AF study and out-with standard routine care at UHCW. The participant will be able to refuse to participate in the study without giving any reasons and this will not affect their standard treatment in any way.

If informed consent is initially obtained remotely e.g., consent statements agreed over the phone, the participant will be asked to counter sign their consent form on their first study visit to the hospital before starting any assessments. A copy of the signed consent form will then be provided to the participant.

The consent procedure will be undertaken by a delegated member of the research team and will involve:

- Discussion between the potential participant and an authorised individual who is knowledgeable about the research and about the nature and objectives of the trial and possible risks associated with their participation.
- Presentation of written material (e.g., information leaflet and consent document which must be approved by the REC and comply with GCP, local regulatory requirements and legal requirements).
- Opportunity for potential participants to ask questions.
- Assessment of capacity. For consent to be ethical and valid in law, participants must be capable of giving consent for themselves. A capable person will:
  - understand the purpose and nature of the research,
  - understand what the research involves, its benefits (or lack of benefits), risks and burdens,
  - understand the alternatives to taking part,
  - be able to retain the information long enough to make an effective decision,
  - be able to make a free choice,
  - be capable of making this specific decision at the time it needs to be made (though their capacity may fluctuate, and they may be capable of making some decisions but not others depending on their complexity),
  - where participants are capable of consenting for themselves but are particularly susceptible to coercion, it is important to explain how their interests will be protected.

Patients who are determined to lack capacity will be excluded from recruitment to the CREED AF study.

- 1. **Blood Tests**

Blood tests are performed as part of standard care for patients who are referred for first-time AF ablation. We will retrospectively collect data that is available on the patient’s hospital record from their pre-ablation blood test following consent. Biomarkers we are interested in including, but are not limited to full blood count, renal function, thyroid function, BNP, and Troponin.

- 1. **Randomisation**

Pre-randomisation eligibility checks will be carried out to ensure that potential participants meet the eligibility criteria and are not randomised in error. Written consent for entry into the trial and baseline assessments must be obtained prior to randomisation. Subjects will be randomised once they have been registered as eligible for randomisation and attended their baseline assessment.

After the baseline assessment, participants will be randomised on a 1:1 basis to the CREED AF intervention or usual care. Participants will be stratified by sex (male or female), AF type (Paroxysmal or Persistent), left ventricular ejection fraction (not impaired ≥50% or impaired <50%), and Amiodarone use (Yes or No).

Randomisation will be carried out using an online validated randomisation sequence generator, as part of the Electronic Data Capture system. The block sizes to be used in the randomisation sequence in the electronic data capture (EDC) system will be determined by the trial statistician prior to setting up the EDC.

Participants will be informed of their allocation to treatment in writing (e.g., email or post), with information on the next steps depending on their allocation.

- 1. **Blinding**

It is not possible to blind several members of the study team as they will directly be involved in the delivery of the prehabilitation and rehabilitation. Protecting our primary outcome measures, the exercise physiologists leading the cardiopulmonary exercise testing at the 10-week post-ablation follow-up will be blinded from the participant’s treatment allocation. We will endeavour to inform patients not to let the physiologists know their treatment allocation when communicating with them. We will record if a participant tells the follow-up exercise physiologist their allocation.

- 1. **Baseline data**

All participants will have the same baseline data collected following consent. This includes, but is not limited to: demographic data, medical history, current medications and details of their AF. All participants will be asked to complete a few validated questionnaires to assess their HRQoL, including the EQ-5D-5L, SF-36 and AFEQT. This initial dataset can be collected over the phone following consenting of the participant into the trial. The questionnaires can be posted to the participant to complete at home and return at a following clinic appointment or they can be emailed out to the participant to complete online using the EDC System.

- 1. **Cardiopulmonary Exercise Testing**

The CPET will be performed by both the control and intervention groups at baseline and 10 weeks (±2 weeks) post-ablation. The test will be used to assess exercise capacity and will give an indicator of the participant’s cardiorespiratory fitness. The tests will be performed at UHCW’s respiratory laboratory. The lab is UKAS accredited to IQIPS standard to perform CPET. The local UHCW standard operating procedure for CPET will be followed for all participants during their VO_2peak_ test. This information is available in full detail in a separate document, but briefly:

1. The participant will have their height and weight measured and information about the current medications collected.
2. They will have the procedure explained to them in full detail and any questions they have will be answered.
3. A spirometry measurement of forced vital capacity will be obtained before the CPET begins.
4. A 12-lead ECG will be attached to the participant.
5. A Hans Rudolph face mask will be fitted to the participant and baseline data collected.
6. A blood pressure cuff and pulse oximeter will be attached with measurements taken at baseline and during the test.
7. The exercise protocol will begin with increasing exercise intensity until the participant feels they cannot continue, when cadence falls by 5% or more or when the individual leading the exercise test requests it to be stopped.
8. Recovery period involving low intensity exercise.
   1. **Standard Care Group**

Participants who are randomised into the control group will complete the outcome assessments at baseline and 10 weeks (±2 weeks) post-ablation. Further HRQoL questionnaires and medical records will be reviewed at 12 months (±4 weeks) post-ablation. They will also receive a 30-minute 1-to-1 education session (remotely or in person) from a CREED AF practitioner before their AF ablation to go through the risk factors of AF and what they can do to improve their health. The session will involve a discussion with the participant around risk factors related to AF and recommendations for what actions the participant should take to minimise these factors, sign-posting to publicly available patient information leaflets (e.g. <https://heartrhythmalliance.org/aa/uk/resources/arrhythmia-alliance-patient-resources>). For the rest of the trial, they will follow the standard care pathway for ablation patients.

- 1. **Intervention Group**

The CREED AF intervention will involve 2 distinct intervention strategies that come together. These strategies are the exercise intervention and the education intervention. Participants who are randomised to this intervention will have an additional consultation with a member of the research team who will go over again in more detail what the CREED AF intervention is, and what it involves for the participant.

Exercise Sessions

*Format*: To ensure generalisability to the NHS, the underpinning framework of the CREED AF exercise intervention is based on UK cardiopulmonary rehabilitation guidelines and service delivery models (62).

*Programme Design*: Participants randomised to the CREED AF intervention can immediately join existing cardiopulmonary rehabilitation exercise programmes rather than waiting for the recruitment of sufficient numbers to form a discrete group of trial participants.

Individual assessment and exercise familiarisation

*Individual assessment*: A 1-to-1 online appointment with a CREED AF ‘practitioner’ (specialist cardiopulmonary clinical exercise physiologist or physiotherapist). Participants will undergo an ‘assessment,’ as per standard practice in UK cardiopulmonary rehabilitation programmes, to assess medical history, medication, and discussion of participant goals, expectations, fears, and concerns.

*Exercise prescription*: The CREED AF practitioner will prescribe a tailored, individualised exercise programme within pre-specified parameters, as outlined in the information sheet. Clinical information, data from the exercise assessment, and patient centred goal setting will be used to devise a safe and effective exercise prescription. There are no accepted guidelines for exercise training in patients due for ablation. Our intervention will be individualised and based on existing evidence in similar populations, our PPI sessions, and our centres’ expertise in the provision of exercise for clinical populations.

*Familiarisation*: Exercise will be delivered on a rolling basis, so a participant can immediately join a pre-existing cardiopulmonary exercise group that is at a similar physical level to them. During the first week of the programme, the clinical staff will assess their capability to carry out the prescribed exercise. Following this, the exercise that the participant is undertaking could be altered slightly to better suit their physical capabilities. The familiarisation period will enable participants to build confidence, whilst CREED AF practitioners refine and optimise the exercise prescription.

Exercise Programme (2-3 times/week for 6-8 weeks, pre- and post-ablation)

Exercise training will be facilitated by a trained CREED AF practitioner using:

1. Participant manual with details of the exercise programme, instruction on safe and effective exercise, and a logbook to self-record completed exercise. These materials will be provided to participants at the start of the intervention.
2. Live online exercise sessions held at the Atrium Health Centre, led by a CREED AF practitioner to allow participants to complete in person exercise sessions with other participants and receive real time instruction and feedback.

Where possible, participants will join pre-existing cardiopulmonary exercise sessions already being run at the Atrium Health Centre, so that participants do not have to wait until a sufficient number of patients are recruited before beginning the intervention. The number of times that a patient needs to attend these sessions each week will be prescribed by the exercise practitioner at the individual assessment stage. There may be a combination of gym-based sessions and ‘live’ online exercise sessions at home, depending on their individual circumstances (e.g., ability to get to the Atrium Health Centre multiple times per week).

For those that are able, moderate intensity dynamic cardiovascular exercise will be performed. In addition, ‘functional fitness training’ will aim to improve general musculoskeletal deconditioning. This type of training is targeted specifically at the components of physical fitness required for activities of daily living, making use of multi-plane motion, to improve agility, co-ordination, proprioception, balance, and functional strength.

Attendance of exercise sessions (both online and in-person) will be monitored throughout the study and a compliance rate calculated at the end of the intervention period (both pre- and post-ablation). A participant will be defined as having completed the prehabilitation intervention if they have a compliance rate of 66.7% (at least 8 sessions out of 12 sessions) or above. Similarly, a compliance rate of 66.7% (at least 8 sessions out of 12 sessions) of rehabilitation intervention is defined as full compliance.

Education Sessions (alternate weeks for 6-8 weeks, pre- and post-ablation)

The 1-to-1 education sessions in the CREED AF Intervention arm will be conducted by an exercise practitioner/researcher and will be performed either online, over the phone, or in-person. The sessions will incorporate comprehensive behavioural change and motivational strategies to improve patient adherence and compliance to risk factor modification with the aim of improving both short- and long-term adherence to exercise and lifestyle modification goals and reduce their exposure to lifestyle risk factors (e.g., smoking, alcohol consumption, weight reduction etc.). This will be guided by a participant workbook, and further sign-posting to support groups will be in accordance to individual participant’s requirements. A full compliance of education sessions is at least 2 sessions (of a total of 3; 66.7%) at each prehabilitation and rehabilitation intervention.

- 1. **Follow-up data**

In addition to cardiopulmonary exercise testing at 10 weeks follow-up post-ablation, all patients will also repeat the baseline questionnaires to assess their HRQoL, including the EQ-5D-5L, SF-36 and AFEQT, at significant intervals of the intervention journey (as outlined in the Schedule of Events). To assess for AF recurrence and burden, 7-day Holter ECG monitoring will be placed on patients at the 10 weeks and 12 months post-ablation follow-up appointments. The 10-week (±2 week) period will be seen as equivalent to a 3-month blanking period that is normally considered post-ablation in which presence of AF is not seen as a recurrence. The definition of an AF recurrence post-ablation will be an arrhythmia with the characteristics of AF that is either captured on a 12-lead ECG or documented to last for at least 30 seconds on ECG monitoring (1). At 12 months post-ablation, a retrospective review of medical records will be completed to collect data for study defined MACE. This will include, but will not be limited to, a review of clinic letters, medical coding, and A&E records.

- 1. **End of Study Definition**

The end of study definition will be when the last participant has completed their 12-month follow-up appointment and data collection post-ablation.

- 1. **Assessment and Management of Risk**

In keeping with UHCW SOPs, a risk assessment will be implemented, focusing on ensuring safe exercise assessment and prescription for people with AF. Primarily this will involve undertaking appropriate pre-exercise screening; an assessment to ensure the participant can take part in the intervention, both at the Atrium Health Centre, and at their home safely whilst also making sure that they can safely take part in the cardiopulmonary exercise tests. Also, that there will be the provision of appropriately trained staff, suitable facilities, remote supervision, and a comprehensive emergency strategy, with thorough clinical procedures for implementation. Participants will be advised initially to have another adult present at home when carrying out exercises as part of the intervention.

CPET will take place in a UKAS accredited IQIPS standard laboratory. A fully trained member of the research team will run these tests. If a participant is identified to be contraindicated to an exercise test, then their eligibility for the study will be reviewed (i.e., whether the contraindication is temporary in preventing an exercise test).

Physical activity carries a very small risk for patients with AF. Physical activity is likely to cause tiredness, breathlessness, and sore muscles however this is to be expected when starting a new exercise programme and should not be treated as adverse events.

The standard ablation procedure will be carried out by qualified staff members at UHCW. Ablation carries a small risk of further complications which are outlined below:

- <1% risk of vascular injury (including needing surgery)
- 1% risk of pericardial effusion/tamponade
- ~1% risk of stroke
- ~1% risk of phrenic nerve injury
- <1% risk of AV block needing pacing
- 0.5% risk of pulmonary vein stenosis
- Risk of needing emergency cardiac surgery (1:400)
- Other small risks: needing blood transfusion

Any of the above events that occur during the CREED AF study following an AF procedure will be monitored by the trial team and recorded as an adverse outcome of ablation. If it becomes apparent that the CREED AF intervention group are experiencing a statistically significant greater number of these events than the control, then this will be raised and reported in line with local Trust SOPs and reported to the REC as appropriate.

# **STATISTICS AND DATA ANALYSIS**

- 1. **Sample Size Calculation**

The sample size calculation was based on the analysis of the change in the primary outcome (VO_2peak_) at 10 weeks post-AF ablation from baseline. Previous studies investigated VO_2peak_ at 6 months post-ablation, which we assume to be similar at 10 weeks post-ablation. Based on Kato *et al.*, we can assume that the VO_2peak_ standard deviation is around 3ml/kg/min at baseline and 4ml/kg/min at 10 weeks post-ablation (63). As the correlation between the time points is unknown, we assume a value of r=0.5. Consequently, the standard deviation of the difference from 10 weeks post-ablation to baseline is around 3.6ml/kg/min. Based on Fiala *et al.* we can assume that the change in VO_2peak_ will be around 2ml/kg/min due to AF ablation and a further 4ml/kg/min (63,64) due to cardiac rehabilitation (which comprises pre- and post-AF ablation rehabilitation). Therefore, the difference between the intervention arm and standard care is expected to be 4ml/kg/min at 10 weeks (±2 weeks) post AF ablation compared with baseline. To detect a conservative difference of 2.5ml/kg/min (an increase in 2.5ml/kg/min VO_2peak_ in the intervention arm from standard care, at 10 weeks post-ablation compared with baseline) at 5% significance level, with 90% power, we require 88 patients in total. To allow a drop-out rate of 20%, we will recruit 106 patients, with 53 randomly allocated to each group.

- 1. **Planned Recruitment Rate**

Participants who are referred for first-time AF ablation at UHCW NHS Trust with AF symptoms and meet the remaining inclusion/exclusion criteria will be eligible for the study. At UHCW we are seeing ~300 patients each year at UHCW for first-time AF ablation and it is predicted that ~200 of these patients will be eligible. We will be aiming to recruit around 1-3 patients per week over an ∼16-month period to recruit the participant population within the ∼16-month recruitment period.

- 1. **Statistical Considerations**

The statistical analysis plan will be finalised prior to primary outcome analysis. It will include a technical and detailed description of the statistical analyses described here. Demographics and baseline characteristics categorical data will be summarised as frequency and percentage, continuous data will be summarised using the following descriptive statistics: frequency (total number of missing and non-missing available for summary), mean and standard deviation (SD), median, 25th and 75th percentiles, minimum and maximum by treatment arm and as overall.

- 1. **Primary outcome analysis**
- Treatment arms: standard care with a 30-minute education with a CREED AF practitioner vs CREED AF intervention including the effects of treatment (including prehabilitation, rehabilitation, and education) discontinuation.
- Target population: all participants with both V0_2peak_ at baseline and 10-week post-AF ablation. Participants assigned to standard care through randomisation will be the control group. Participants assigned to CREED AF intervention will be the active intervention group.
- Endpoints: VO_2peak_ at baseline and 10-week post-AF ablation.
- Handling of intercurrent events: treatment discontinuation due to any reason is addressed as treatment policy strategy and all follow-up data will be included in the analysis. Death is the most serious adverse event and missing follow-up data will be excluded from analysis and set to missing. No other intercurrent events are anticipated.
- Population-level summary measure: difference in means between treatment arms by fitting linear regression model where the VO_2peak_ at 10-week post-AF ablation is the response and the predictors are the VO_2peak_ at baseline and treatment arm. We will also adjust for other baseline measurements, e.g., sex and Amiodarone use.

Note that the primary endpoint analysis will be mainly performed by co-investigator NC as part of the requirement to fulfil his PhD. Hence, this will be presented before the long-term 12-month follow-up is complete and analysed.

- 1. **Secondary outcome analysis**

All the scoring of HRQoL (EQ-5D-5L, SF-36 and AFEQT) will be computed as per the instrument algorithm. The scores will then be summarised descriptively overall and by treatment arms at baseline and the short- and long-term follow-up time points. The change of HRQoL from baseline to 10-week, from baseline to 12-month post-ablation, and the difference between treatment arms will be estimated with their associated 95% confidence intervals (CIs). We may explore the longitudinal effect of long-term HRQoL (12-month post-ablation). Proportion of significant AF event by treatment arms will be estimated with associated 95% CI using Kaplan-Meier. Similarly, the proportion of individual and composite MACE and its associated 95% CI by treatment arms will be estimated. The recurrence of AF burden from the Holter and each of the MACE occurrence will also be estimated by treatment arms as a time-to-event estimand. We will also perform a subgroup analysis by types of AF (persistent and paroxysmal AF) for all endpoints. We may also explore for any potential confounders in VO_2peak_ within the study, and the complier averaged causal effect (CACE) analysis as a sensitivity analysis.

- 1. **Procedure(s) to account for missing or spurious data**

We will endeavour to collect all data possible from each participant during their enrolment in the study. Data monitoring will be carried out by the trial manager/researcher and any missing data points will be raised as a query on the online database which will be followed up by the delivery team. If it is judged that the same critical data points (as outlined in the data management plan) are consistently being missed (>10%) then this will be raised at the following TMG for corrective action.

Multiple imputation methods will be used as secondary analysis if more than 10% of the outcome variable is missing.

- 1. **Economic Evaluation**

To assess the costs, benefits, and overall cost-effectiveness of the intervention, we will integrate a prospective economic evaluation into the trial. The time horizon of the analysis will be one year, congruently with the duration of the trial. The primary perspective will be NHS and Personal Social Services (PSS), with secondary analyses including additional patient costs accruing over the 12 months period post ablation.

Resource use and costs

Intervention resource use and costs associated with implementing and delivering the intervention will be captured within the trial. For example, for both arms we will record and then cost the AF cryoballoon and radiofrequency ablation procedure using published costing resources such as the NICE reference costs (40,41). For the intervention arm we will additionally record and cost the resource use associated with attendance to the pre- and post-AF ablation rehabilitation intervention components. Further use of other NHS care (e.g. primary care appointments, outpatient visits, inpatient admissions) and related costs incurred by the patient (e.g. out-of-pocket expenses and time-related costs) will be captured using a resource use measure (RUM) at different points in time. To cost the intervention and healthcare resource use, we will apply unit costs using national reference sources, e.g., PSSRU (65). This will allow the calculation of total healthcare costs related to AF from baseline to 12 months for both arms of the trial including those attributable to the intervention.

Outcomes

Responses to EQ-5D-5L, a widely used preference-based quality of life instrument, collected at baseline and follow-up visits will be used to calculate quality-adjusted life years (QALYs) using UK value set recommended by NICE at the time of the analysis. Similarly, responses to the SF-36 questionnaire will be used to derive preference-based quality of life indices through the SF-6D algorithm (66). Baseline utility scores will be used both in the construction of QALYs and as an adjusting covariate within the regression analysis. Mechanisms of missingness of data will be explored, and multiple imputation will be used if missing data is prevalent.

Analysis

The analysis will be conducted on an ‘intention to treat’ basis. Missing data will be accounted for by using appropriate techniques, such as multiple imputation, depending on the extent and type of missing items. As the distribution of cost is usually skewed by the existence of patients with very high costs, the calculated mean per-patient cost will be given alongside confidence intervals obtained through non-parametric bootstrap methods (67). Incremental analysis will be undertaken to calculate the difference in costs and the difference in outcomes (QALYs) associated with the intervention. Results will be presented in the form of incremental cost-effectiveness ratios (ICER), reflecting the extra cost for an additional unit of outcome. Sensitivity analyses will be undertaken to assess the impact of different assumptions on the results (68). To account for the inherent uncertainty due to sampling variation, the joint distribution of differences in cost and outcomes (QALYs) will be derived by carrying out a large number of non-parametric bootstrap simulations (69).

The simulated cost and outcome pairs will be depicted on a cost-effectiveness plane and will be plotted as cost-effectiveness acceptability curves (CEACs) (70). CEACs will show the probability of CREED AF being cost-effective across a range of possible values of willingness to pay for an additional QALY.

# **DATA MANAGEMENT**

- 1. **Data Collection and Management**

Trial data will be collected on CRFs and validated questionnaires, either on paper or electronically. An online validated, GCP compliant, Electronic Data Capture system will be used to record and store trial data. Individual user log-in access to this database will be granted to only those in the study team that require it for the performance of their role.

Screening and recruitment logs of all patients approached to take part, and participants enrolled in the trial will be held at each site. Upon confirmation of eligibility, participants will be assigned a unique trial ID, which will be used to identify all documents associated with that participant for the duration of the trial.

Participants will be also able to complete the outcome questionnaires by post or online if they prefer. If done online, they will be emailed a secure, unique link to complete the questionnaires, where their answers will directly input onto the database.

Following all query resolution, the database will be exported for statistical analysis. These procedures along with data entry instructions will be detailed in a Data Management Plan (DMP) produced by the trial manager and co-investigators.

- 1. **Access to Data**

Personal data collected during the trial will be handled and stored in accordance with the GDPR. Disclosure of confidential information will only be considered if there is an issue which may jeopardise the safety of the participant or another person, according to UHCW SOPs and the UK regulatory framework. There is no reason to expect this situation to occur in this trial more than any other.

All data will be pseudonymised after the collection of baseline demographics for each participant. Confidentiality will be strictly maintained, and names or addresses will not be disclosed to anyone other than the staff involved in running the trial. All electronic participant-identifiable information will be held on a secure, password-protected database accessible only to essential personnel. Paper forms with participant-identifiable information will be held in secure, locked filing cabinets within a restricted area. Participants will be identified by a unique trial ID only.

Direct access will be granted to authorised representatives from the Sponsor, host institution and the regulatory authorities to permit trial-related monitoring, audits, and inspections - in line with participant consent.

Requests for data sharing will be managed in accordance with UHCW policy on data sharing. The datasets generated during and/or analysed during the current study are/will be available upon request after publication of the main study results. The publication of a trial protocol, trial results and trial data will be in line with UHCW SOPs.

- 1. **Archiving**

Following the resolution of queries and confirmation of study close-out by the Chief Investigator, all essential documentation will be transferred to a third-party archiving service, which provides suitable fire and water-resistant facilities. Study files will be archived for a period of 10 years. Access to the study documentation will be restricted to named individuals within the study team with express permission from the Chief Investigator.

# **TRIAL OVERSIGHT**

- 1. **Role and Responsibilities of the Sponsor**

UHCW has agreed to act as sponsor for this trial and will undertake the responsibilities of sponsor as defined by the UK Policy Framework for Health and Social Care Research and ICH Good Clinical Practice. An authorised representative of the Sponsor has approved the final version of this protocol with respect to the trial design, conduct, data analysis and interpretation and plans for publication and dissemination of results. As sponsor, UHCW provides indemnity for this trial and, as such, will be responsible for claims for any negligent harm suffered by anyone as a result of participating in this trial. The indemnity is renewed on an annual basis and will continue for the duration of this trial.

- 1. **Role and Responsibilities of the Funder**

Funding for this trial is provided by Boston Scientific. The design and management of this trial are entirely independent of the funder.

- 1. **Trial Management Arrangements**
     1. **Trial Coordinator/Manager**

The Trial Coordinator/Manager will have responsibility for overseeing day to day coordination of the trial and reporting regularly to the TMG. The Trial Coordinator/Manager’s responsibilities include, but are not limited to:

- Coordinating protocol development, patient, and trial management documents
- Correspondence with study funder and tracking of progress against agreed milestones
- Setting up and maintaining the Trial Master File;
- Ensuring necessary approvals are in place before the start of the trial at each site;
- Providing training to trial personnel;
- Providing data management support; including data input, maintenance of the trial database and raising of queries
- Producing trial progress reports and coordinating TSC meetings and minutes;
- Ensuring data security and quality and ensuring data protection laws are adhered to;
- Ensuring complete records are in place for audit and monitoring purposes;
- Ensuring the trial is conducted in accordance with the ICH GCP;
- Archiving all original trial documents including the data forms in line with UHCW NHS Trust policy
  - 1. **Principal Investigators**

Site Principal Investigator responsibilities include, but are not limited to:

- Ensuring that the trial is conducted as set out in the protocol and supporting documents
- Delegating trial related responsibilities only to suitably trained and qualified personnel and ensuring that those with delegated responsibilities fully understand and agree to the duties being delegated to them;
- Ensuring that CVs and evidence of appropriate training for all Site staff are available in the Trial Site File
- Ensuring that all delegated duties are captured in the study Delegation Log
- Ensuring all Adverse Events are documented and reported promptly to the Trial Manager;
- Accountability for trial treatments at their site;
- Ensuring the trial is conducted in accordance with ICH GCP principles;
- Allowing access to source data for monitoring, audit and inspection;
- Ensuring that all source data is complete and provided to the Trial Manager at regular intervals
  - 1. **Trial Management Group (TMG)**

The Trial Management Group, consisting of the project staff and co-investigators involved in the day-to-day running of the trial, will meet regularly throughout the trial.

# **ADVERSE EVENT MANAGEMENT**

- 1. **Adverse Events**

An Adverse Event (AE) is defined as any untoward medical occurrence involving a participant, which does not necessarily have a causal relationship with the intervention or trial.

**Expected** **AEs,** related to the exercise outcome assessments or the exercise intervention, include ‘normal’ levels (for the individual) of:

- Breathlessness
- light headedness/dizziness
- muscle and joint stiffness/soreness
- tiredness/fatigue

There are a few AEs which are expected following AF ablation and occur after roughly 2-4% of all AF ablation cases. These AEs are listed below:

- Vascular injury
- Pericardial effusion/tamponade
- Stroke/Transient Ischaemic Attack
- Myocardial Infarction
- Phrenic nerve injury
- Atrioventricular block
- Pulmonary vein stenosis

Of these conditions there is a small risk (1:400) of requiring further cardiac surgery or a blood transfusion. Each AE will be assessed by the CI or a delegated clinician to assess causality to the CREED AF intervention. In the unlikely event that it is decided that one of the above post AF ablation conditions is related to the CREED AF intervention this will be reported as such in line with local Trust policy and national safety reporting guidelines.

Recording procedures will be the same for both trial groups. Expected AEs will be recorded on the CRF (AE form).

**Unexpected AEs** related to the exercise outcome assessments or the exercise intervention for both the intervention and usual care groups, will be recorded on the appropriate CRF (AE form) and returned routinely to UHCW. Unexpected AEs will be logged and reported at TMG meetings. Should multiple of the same unexpected AE occur during the trial period then this will trigger the TMG to review the study procedures to see if there are any changes that need to be implemented.

AEs in the intervention group will be determined through patient report at either their in-person intervention session, exercise outcome assessment (CPET) or via their 1-to-1 appointments with the research team (online, in person or over the phone), including a follow-up phone call the day after the outcomes assessment (VO_2peak_) appointments until resolution of the event. AEs in the standard care group will be determined via a check of their medical records, on the day of their exercise outcome session (CPET), and a follow-up phone call the day after the outcomes assessment (VO_2peak_) appointments until resolution of the event.

- 1. **Serious Adverse Events (SAEs)**

Over the study period, people may be admitted to hospital for reasons other than their AF ablation procedure. Admissions data will be collected from self-report and via medical records checks.

For CREED AF, an SAE will be an untoward medical occurrence that fulfils one or more of the following criteria:

- Results in death
- Is life-threatening
- Requires hospitalisation or prolongation of existing hospitalisation
- Results in persistent or significant disability or incapacity
- Requires medical intervention to prevent one of the above, or is otherwise considered medically significant by the investigator

All admissions to hospital are an SAE. The following SAEs are expected with CREED AF and **do not** require additional reporting for this trial, but must be recorded in the relevant section(s) of the CRF:

- Disease progression: worsening symptoms/AF attrition unrelated to the exercise intervention.
- Treatment, which was elective or pre-planned, for a pre-existing condition, not associated with any deterioration in condition.
- General care, not associated with any deterioration in condition.

**Reportable SAEs - intervention group:** SAEs related to exercise sessions (remote or group sessions within the Atrium Health Centre), or outcomes assessments (VO_2peak_) are possible. In the intervention group, any event that occurs at any time (remote or group sessions within the Atrium Health Centre,) between baseline outcomes assessment and the 10 weeks (±2 weeks) post-AF ablation, or within 24 hours of the outcome assessment appointments (VO_2peak_), will be recorded and reviewed by the chief investigator to determine if it is directly attributable to the intervention, and investigated in line with UHCW’s SOP. This will be determined by participant report at each intervention session or 1-to-1 education session and a follow-up phone call the day after the outcomes assessment (VO_2peak_) appointments until resolution of the event.

If a participant has not attended two consecutive in-person intervention appointments at the Atrium Health Centre, without informing a member of the CREED AF study team, their status will be checked on local electronic clinical records by the local CREED AF clinical rehabilitation team (with participant consent). If their status is unclear, the CREED AF clinical rehabilitation team will attempt to make contact on the phone at least three times.

**Reportable SAEs - standard care group**: any event occurring within 24 hours of each of the assessment appointments (baseline and 10 weeks (±2 weeks) post ablation) will be recorded and reviewed to determine if it is directly attributable to the trial; this will be determined via a phone call to the participant the day following their appointment.

All participants experiencing SAEs during the period up to the 12-month assessments or during the 24-hour periods after the baseline and 10 weeks (±2 weeks) post-AF ablation outcomes assessment (VO_2peak_) will be followed-up until resolution of the event.

The causality of SAEs (i.e. relationship to trial intervention) will be assessed by the investigator(s) using the SAE form (Table 2).

*Table 2: SAE Causal relationship*

| **Relationship**  **to trial intervention** | **Description** |
| --- | --- |
| Unrelated | There is no evidence of any causal relationship |
| Unlikely to be related | There is little evidence to suggest there is a causal relationship (e.g. the event did not occur within a reasonable time after administration of the trial intervention). There is another reasonable explanation for the event (e.g. the patient’s clinical condition, other concomitant treatment). |
| Possible relationship | There is some evidence to suggest a causal relationship (e.g. because the event occurs within a reasonable time after administration of the trial intervention). However, the influence of other factors may have contributed to the event (e.g. the patient’s clinical condition, other concomitant treatments). |
| Probable relationship | There is evidence to suggest a causal relationship and the influence of other factors is unlikely. |
| Definitely related | There is clear evidence to suggest a causal relationship and other possible contributing factors can be ruled out. |

To establish causality, the following information should be collected for each SAE:

- full details in medical terms and case description
- event duration (start and end dates, if applicable)
- action taken
- outcome
- seriousness criteria
- causality (i.e. relatedness to intervention), in the opinion of the PI
- whether the event would be considered expected or unexpected

SAEs that are deemed to be unexpected and possibly, probably or definitely related to the trial interventions or outcomes assessments, will be notified to the Research Ethics Committee (REC) within 15 days. All such events will be reported to the TMG at their next meeting.

Any change of condition or other follow-up information should be communicated to the Sponsor as soon as it is available or at least within 24 hours of the information becoming available. Events will be followed until resolution or a final outcome has been reached. A member of the PI’s trial team will be instructed to closely monitor each participant who experiences a SAE, until the outcome of the SAE has been determined.

- 1. **Responsibilities**

*Chief Investigator/delegate or independent clinical reviewer:*

- Clinical oversight of the safety of patients participating in the trial, including an ongoing review of the risk / benefit.
- Using clinical judgement in assigning seriousness and causality of SAEs where it has not been possible to obtain local medical assessment.
- Using clinical judgement in assigning expectedness.
- Immediate review of all related and unexpected SAEs.
- Review of specific SAEs in accordance with the trial risk assessment and protocol as detailed in the Trial Monitoring Plan.
- Production and submission of annual reports to the relevant REC.

*Sponsor:*

- Central data collection and verification of AEs and SAEs, according to the trial protocol.
- Delegating individuals to perform SAE causality and expectedness assessments on behalf of the Sponsor.
- Reporting safety information to the CI, delegate, or independent clinical reviewer for the ongoing assessment of the risk/benefit according to the Trial Monitoring Plan.
- Expedited reporting of related and unexpected SAEs to the REC within required timelines.
- Notifying Investigators of related and unexpected SAEs that occur within the trial.

*Trial Management Group:*

- Periodically reviewing safety data.

# **MONITORING, AUDIT & INSPECTION**

The study may be monitored by the Research & Development Department at UHCW as representatives of the Sponsor, to ensure that the study is being conducted as per protocol, adhering to Research Governance and GCP. The approach to, and extent of, monitoring may be specified in a trial monitoring plan determined by the risk assessment undertaken prior to the start of the study.

# **ETHICAL AND REGULATORY CONSIDERATIONS**

- 1. **Ethical Approval and Research Governance**

The study will be conducted in compliance with the principles of the ICH GCP guidelines and in accordance with all applicable regulatory guidance, including, but not limited to, the UK policy framework for health and social care research. Ethical approval for this study will be sought from the Research Ethics Committee combined with Health Research Authority (HRA) approval. No study activities will commence until favourable ethical opinion and HRA approval has been obtained. Progress reports and a final report at the conclusion of the trial will be submitted to the approving REC within the timelines defined by the committee. Confirmation of capacity and capability will be obtained from the R&D department prior to commencement of the study at all participating sites.

- 1. **Notification of Serious Breaches to GCP and/or Trial Protocol**

A “serious breach” is a breach which is likely to effect, to a significant degree:

1. the safety or physical or mental integrity of the participants of the trial;
2. the scientific value of the trial

If a serious breach occurs, the sponsor will be notified immediately of any case where the above definition applies during the trial conduct phase.

- 1. **Peer Review**

Prior to submission to the research ethics committee, the study protocol and other patient facing documents have been reviewed by expert members in the field of cardiology and ablation.

- 1. **Public and Patient Involvement**

The study was presented to the Patient and Public Research Advisory Group (PPRAG) at UHCW to receive expert lay feedback on the study design and methodology. Following engagement with the PPRAG at UHCW they were supportive of this project. They recommended that people who do not have access to video conferencing software (smartphones or webcams) be excluded due to the reliance on the live exercise sessions at home in the CREED AF intervention. The group were also happy to see the familiarisation period incorporated into the programme.

- 1. **Data Protection and Patient Confidentiality**

The study will comply with the current Data Protection regulations and regular checks and monitoring will be undertaken by the Trial Manger to ensure compliance. Participants will be assigned a unique identifier upon enrolment into the study to allow pseudonymisation of patient-identifiable data. Access to patient identifiable data will be restricted to members of the study co-ordination team who require it for the performance of their role. Electronic data will be stored on password protected encrypted drives and hard copies of study documents will be stored in locked filing cabinets in secure entry-card protected sites.

# **DISSEMINATION POLICY**

Results of the trial will be prepared by the research team and lay partners and submitted to funders as a final report. Findings will be submitted to peer-reviewed journals and disseminated to the medical and exercise rehabilitation communities. Papers will be published in open-access journals describing the development of the CREED AF intervention, the trial protocol, and results and data, in accordance with recommended guidance for transparent reporting, the CONSORT guidelines (www.consort-statement.org) and UHCW SOPs: Publication & Dissemination. Abstracts will also be submitted to national and international conferences.

# **REFERENCES**

1. Calkins H, Hindricks G, Cappato R, Kim Y-H, Saad EB, Aguinaga L, et al. 2017 HRS/EHRA/ECAS/APHRS/SOLAECE expert consensus statement on catheter and surgical ablation of atrial fibrillation. Heart Rhythm. 2017 Oct;14(10):e275–444.

2. Theodoreson MD, Chohan BC, McAloon CJ, Sandhu A, Lancaster CJ, Yusuf S, et al. Same-day cardiac catheter ablation is safe and cost-effective: Experience from a UK tertiary center. Heart Rhythm. 2015 Aug 1;12(8):1756–61.

3. He H, Datla S, Weight N, Raza S, Lachlan T, Aldhoon B, Panikker S, Dhanjal T, Yusuf S, Foster W, Hayat S, Osman F. Safety and cost-effectiveness of same-day complex left atrial ablation. International Journal of Cardiology 2021; 322: 170–174. https://doi.org/10.1016/j.ijcard.2020.09.0664

4. Skelly A, Hashimoto R, Al-Khatib S, Sanders-Schmidler G, Fu R, Brodt E, et al. Catheter Ablation for Treatment of Atrial Fibrillation [Internet]. Rockville (MD): Agency for Healthcare Research and Quality (US); 2015 [cited 2020 Jan 24]. (AHRQ Technology Assessments). Available from: http://www.ncbi.nlm.nih.gov/books/NBK305760/

5. Overview | Atrial fibrillation: management | Guidance | NICE [Internet]. [cited 2020 Jan 6]. Available from: https://www.nice.org.uk/guidance/cg180

6. Chugh SS, Havmoeller R, Narayanan K, Singh D, Rienstra M, Benjamin EJ, et al. Worldwide epidemiology of atrial fibrillation: a Global Burden of Disease 2010 Study. Circulation. 2014 Feb 25;129(8):837–47.

7. Colilla S, Crow A, Petkun W, Singer DE, Simon T, Liu X. Estimates of current and future incidence and prevalence of atrial fibrillation in the U.S. adult population. Am J Cardiol. 2013 Oct 15;112(8):1142–7.

8. Kirchhof P, Benussi S, Kotecha D, Ahlsson A, Atar D, Casadei B, et al. 2016 ESC Guidelines for the management of atrial fibrillation developed in collaboration with EACTS. Eur Heart J. 2016 07;37(38):2893–962.

9. Björck S, Palaszewski B, Friberg L, Bergfeldt L. Atrial fibrillation, stroke risk, and warfarin therapy revisited: a population-based study. Stroke. 2013 Nov;44(11):3103–8.

10. Haim M, Hoshen M, Reges O, Rabi Y, Balicer R, Leibowitz M. Prospective National Study of the Prevalence, Incidence, Management and Outcome of a Large Contemporary Cohort of Patients With Incident Non‐Valvular Atrial Fibrillation. J Am Heart Assoc Cardiovasc Cerebrovasc Dis [Internet]. 2015 Jan 21 [cited 2019 Oct 22];4(1). Available from: https://www.ncbi.nlm.nih.gov/pmc/articles/PMC4330072/

11. Krijthe BP, Kunst A, Benjamin EJ, Lip GYH, Franco OH, Hofman A, et al. Projections on the number of individuals with atrial fibrillation in the European Union, from 2000 to 2060. Eur Heart J. 2013 Sep;34(35):2746–51.

12. Benjamin EJ, Wolf PA, D’Agostino RB, Silbershatz H, Kannel WB, Levy D. Impact of atrial fibrillation on the risk of death: the Framingham Heart Study. Circulation. 1998 Sep 8;98(10):946–52.

13. Krahn AD, Manfreda J, Tate RB, Mathewson FA, Cuddy TE. The natural history of atrial fibrillation: incidence, risk factors, and prognosis in the Manitoba Follow-Up Study. Am J Med. 1995 May;98(5):476–84.

14. Nieuwlaat R, Capucci A, Camm AJ, Olsson SB, Andresen D, Davies DW, et al. Atrial fibrillation management: a prospective survey in ESC member countries: the Euro Heart Survey on Atrial Fibrillation. Eur Heart J. 2005 Nov;26(22):2422–34.

15. Chao T-F, Liu C-J, Chen S-J, Wang K-L, Lin Y-J, Chang S-L, et al. CHADS2 score and risk of new-onset atrial fibrillation: a nationwide cohort study in Taiwan. Int J Cardiol. 2013 Sep 30;168(2):1360–3.

16. Schnabel RB, Yin X, PhilimonGona, Larson MG, Beiser AS, McManus DD, et al. Fifty-Year Trends in Atrial Fibrillation Prevalence, Incidence, Risk Factors, and Mortality in the Community. Lancet Lond Engl. 2015 Jul 11;386(9989):154–62.

17. Kerr CR, Humphries KH, Talajic M, Klein GJ, Connolly SJ, Green M, et al. Progression to chronic atrial fibrillation after the initial diagnosis of paroxysmal atrial fibrillation: results from the Canadian Registry of Atrial Fibrillation. Am Heart J. 2005 Mar;149(3):489–96.

18. Andrade J, Khairy P, Dobrev D, Nattel S. The clinical profile and pathophysiology of atrial fibrillation: relationships among clinical features, epidemiology, and mechanisms. Circ Res. 2014 Apr 25;114(9):1453–68.

19. Nattel S, Guasch E, Savelieva I, Cosio FG, Valverde I, Halperin JL, et al. Early management of atrial fibrillation to prevent cardiovascular complications. Eur Heart J. 2014 Jun 7;35(22):1448–56.

20. Dorian P, Jung W, Newman D, Paquette M, Wood K, Ayers GM, et al. The impairment of health-related quality of life in patients with intermittent atrial fibrillation: implications for the assessment of investigational therapy. J Am Coll Cardiol. 2000 Oct;36(4):1303–9.

21. Steinberg BA, Kim S, Fonarow GC, Thomas L, Ansell J, Kowey PR, et al. Drivers of hospitalization for patients with atrial fibrillation: Results from the Outcomes Registry for Better Informed Treatment of Atrial Fibrillation (ORBIT-AF). Am Heart J. 2014 May;167(5):735-742.e2.

22. Healey JS, Oldgren J, Ezekowitz M, Zhu J, Pais P, Wang J, et al. Occurrence of death and stroke in patients in 47 countries 1 year after presenting with atrial fibrillation: a cohort study. Lancet Lond Engl. 2016 17;388(10050):1161–9.

23. European Heart Rhythm Association, European Association for Cardio-Thoracic Surgery, Camm AJ, Kirchhof P, Lip GYH, Schotten U, et al. Guidelines for the management of atrial fibrillation: the Task Force for the Management of Atrial Fibrillation of the European Society of Cardiology (ESC). Eur Heart J. 2010 Oct;31(19):2369–429.

24. Camm AJ, Lip GYH, De Caterina R, Savelieva I, Atar D, Hohnloser SH, et al. 2012 focused update of the ESC Guidelines for the management of atrial fibrillation: an update of the 2010 ESC Guidelines for the management of atrial fibrillation. Developed with the special contribution of the European Heart Rhythm Association. Eur Heart J. 2012 Nov;33(21):2719–47.

25. January CT, Wann LS, Alpert JS, Calkins H, Cigarroa JE, Cleveland JC, et al. 2014 AHA/ACC/HRS guideline for the management of patients with atrial fibrillation: a report of the American College of Cardiology/American Heart Association Task Force on practice guidelines and the Heart Rhythm Society. Circulation. 2014 Dec 2;130(23):e199-267.

26. Hendriks JML, de Wit R, Crijns HJGM, Vrijhoef HJM, Prins MH, Pisters R, et al. Nurse-led care vs. usual care for patients with atrial fibrillation: results of a randomized trial of integrated chronic care vs. routine clinical care in ambulatory patients with atrial fibrillation. Eur Heart J. 2012 Nov;33(21):2692–9.

27. Hendriks JML, Vrijhoef HJM, Crijns HJGM, Brunner-La Rocca HP. The effect of a nurse-led integrated chronic care approach on quality of life in patients with atrial fibrillation. Eur Eur Pacing Arrhythm Card Electrophysiol J Work Groups Card Pacing Arrhythm Card Cell Electrophysiol Eur Soc Cardiol. 2014 Apr;16(4):491–9.

28. Rush KL, Burton L, Schaab K, Lukey A. The impact of nurse-led atrial fibrillation clinics on patient and healthcare outcomes: a systematic mixed studies review. Eur J Cardiovasc Nurs J Work Group Cardiovasc Nurs Eur Soc Cardiol. 2019 Oct;18(7):526–33.

29. Hindricks G, Potpara T, Dagres N, Arbelo E, Bax JJ, Blomström-Lundqvist C, Boriani G, Castella M, Dan GA, Dilaveris PE, Fauchier L, Filippatos G, Kalman JM, La Meir M, Lane DA, Lebeau JP, Lettino M, Lip GYH, Pinto FJ, Thomas GN, Valgimigli M, Van Gelder IC, Van Putte BP, Watkins CL; ESC Scientific Document Group. 2020 ESC Guidelines for the diagnosis and management of atrial fibrillation developed in collaboration with the European Association for Cardio-Thoracic Surgery (EACTS): The Task Force for the diagnosis and management of atrial fibrillation of the European Society of Cardiology (ESC) Developed with the special contribution of the European Heart Rhythm Association (EHRA) of the ESC. Eur Heart J. 2021 Feb 1;42(5):373-498. doi: 10.1093/eurheartj/ehaa612. Erratum in: Eur Heart J. 2021 Feb 1;42(5):507. Erratum in: Eur Heart J. 2021 Feb 1;42(5):546-547. Erratum in: Eur Heart J. 2021 Oct 21;42(40):4194. PMID: 32860505.

30. Soliman EZ, Safford MM, Muntner P, Khodneva Y, Dawood FZ, Zakai NA, et al. Atrial fibrillation and the risk of myocardial infarction. JAMA Intern Med. 2014 Jan;174(1):107–14.

31. Wyse DG, Van Gelder IC, Ellinor PT, Go AS, Kalman JM, Narayan SM, et al. Lone Atrial Fibrillation: Does It Exist? A “White Paper” of the Journal of the American College of Cardiology. J Am Coll Cardiol. 2014 May 6;63(17):1715–23.

32. Anderson L, Oldridge N, Thompson DR, Zwisler A-D, Rees K, Martin N, et al. Exercise-Based Cardiac Rehabilitation for Coronary Heart Disease: Cochrane Systematic Review and Meta-Analysis. J Am Coll Cardiol. 2016 Jan 5;67(1):1–12.

33. Smart NA, King N, Lambert JD, Pearson MJ, Campbell JL, Risom SS, et al. Exercise-based cardiac rehabilitation improves exercise capacity and health-related quality of life in people with atrial fibrillation: a systematic review and meta-analysis of randomised and non-randomised trials. Open Heart [Internet]. 2018 Dec 1 [cited 2019 Dec 17];5(2). Available from: https://openheart.bmj.com/content/5/2/e000880

34. Risom SS, Zwisler A, Johansen PP, Sibilitz KL, Lindschou J, Gluud C, et al. Exercise‐based cardiac rehabilitation for adults with atrial fibrillation. Cochrane Database Syst Rev [Internet]. 2017 Feb 9 [cited 2019 Oct 21];2017(2). Available from: https://www.ncbi.nlm.nih.gov/pmc/articles/PMC6464537/

35. Reed JL, Terada T, Chirico D, Prince SA, Pipe AL. The Effects of Cardiac Rehabilitation in Patients With Atrial Fibrillation: A Systematic Review. Can J Cardiol. 2018;34(10 Suppl 2):S284–95.

36. Cheema FN, Abraham NS, Berger DH, Albo D, Taffet GE, Naik AD. Novel approaches to perioperative assessment and intervention may improve long-term outcomes after colorectal cancer resection in older adults. Ann Surg. 2011 May;253(5):867–74.

37. Silver JK, Baima J. Cancer prehabilitation: an opportunity to decrease treatment-related morbidity, increase cancer treatment options, and improve physical and psychological health outcomes. Am J Phys Med Rehabil. 2013 Aug;92(8):715–27.

38. Hughes MJ, Hackney RJ, Lamb PJ, Wigmore SJ, Christopher Deans DA, Skipworth RJE. Prehabilitation Before Major Abdominal Surgery: A Systematic Review and Meta-analysis. World J Surg. 2019;43(7):1661–8.

39. Wynter-Blyth V, Moorthy K. Prehabilitation: preparing patients for surgery. BMJ [Internet]. 2017 Aug 8 [cited 2020 Jan 23];358. Available from: https://www.bmj.com/content/358/bmj.j3702

40. Giles C, Cummins S. Prehabilitation before cancer treatment. BMJ [Internet]. 2019 Aug 14 [cited 2020 Jan 23];366. Available from: https://www.bmj.com/content/366/bmj.l5120

41. Li C, Carli F, Lee L, Charlebois P, Stein B, Liberman AS, et al. Impact of a trimodal prehabilitation program on functional recovery after colorectal cancer surgery: a pilot study. Surg Endosc. 2013 Apr;27(4):1072–82.

42. Joung B. Risk Factor Management for Atrial Fibrillation. Korean Circ J. 2019 Sep;49(9):794–807.

43. Huxley RR, Lopez FL, Folsom AR, Agarwal SK, Loehr LR, Soliman EZ, et al. Absolute and attributable risks of atrial fibrillation in relation to optimal and borderline risk factors: the Atherosclerosis Risk in Communities (ARIC) study. Circulation. 2011 Apr 12;123(14):1501–8.

44. Lau DH, Nattel S, Kalman JM, Sanders P. Modifiable Risk Factors and Atrial Fibrillation. Circulation. 2017 Aug 8;136(6):583–96.

45. Miller JD, Aronis KN, Chrispin J, Patil KD, Marine JE, Martin SS, et al. Obesity, Exercise, Obstructive Sleep Apnea, and Modifiable Atherosclerotic Cardiovascular Disease Risk Factors in Atrial Fibrillation. J Am Coll Cardiol. 2015 Dec 29;66(25):2899–906.

46. Chamberlain AM, Agarwal SK, Folsom AR, Soliman EZ, Chambless LE, Crow R, et al. A Clinical Risk Score for Atrial Fibrillation in a Biracial Prospective Cohort (from the Atherosclerosis Risk In Communities [ARIC] Study). Am J Cardiol. 2011 Jan 1;107(1):85–91.

47. Abed HS, Wittert GA. Obesity and atrial fibrillation. Obes Rev Off J Int Assoc Study Obes. 2013 Nov;14(11):929–38.

48. Wang TJ, Parise H, Levy D, D’Agostino RB, Wolf PA, Vasan RS, et al. Obesity and the risk of new-onset atrial fibrillation. JAMA. 2004 Nov 24;292(20):2471–7.

49. Wong CX, Sullivan T, Sun MT, Mahajan R, Pathak RK, Middeldorp M, et al. Obesity and the Risk of Incident, Post-Operative, and Post-Ablation Atrial Fibrillation: A Meta-Analysis of 626,603 Individuals in 51 Studies. JACC Clin Electrophysiol. 2015 Jun;1(3):139–52.

50. Heeringa J, Kors JA, Hofman A, van Rooij FJA, Witteman JCM. Cigarette smoking and risk of atrial fibrillation: the Rotterdam Study. Am Heart J. 2008 Dec;156(6):1163–9.

51. Smoking and incidence of atrial fibrillation: results from the Atherosclerosis Risk in Communities (ARIC) study. - PubMed - NCBI [Internet]. [cited 2019 Dec 17]. Available from: https://www.ncbi.nlm.nih.gov/pubmed/21419237/

52. Ettinger PO, Wu CF, De La Cruz C, Weisse AB, Ahmed SS, Regan TJ. Arrhythmias and the ‘Holiday Heart’: alcohol-associated cardiac rhythm disorders. Am Heart J. 1978 May;95(5):555–62.

53. Kodama S, Saito K, Tanaka S, Horikawa C, Saito A, Heianza Y, et al. Alcohol consumption and risk of atrial fibrillation: a meta-analysis. J Am Coll Cardiol. 2011 Jan 25;57(4):427–36.

54. Larsson SC, Drca N, Wolk A. Alcohol consumption and risk of atrial fibrillation: a prospective study and dose-response meta-analysis. J Am Coll Cardiol. 2014 Jul 22;64(3):281–9.

55. Djoussé L, Levy D, Benjamin EJ, Blease SJ, Russ A, Larson MG, et al. Long-term alcohol consumption and the risk of atrial fibrillation in the Framingham Study. Am J Cardiol. 2004 Mar 15;93(6):710–3.

56. Voskoboinik A, Kalman JM, De Silva A, Nicholls T, Costello B, Nanayakkara S, et al. Alcohol Abstinence in Drinkers with Atrial Fibrillation. N Engl J Med. 2020 Jan 2;382(1):20–8.

57. Somers VK, White DP, Amin R, Abraham WT, Costa F, Culebras A, et al. Sleep apnea and cardiovascular disease: an American Heart Association/american College Of Cardiology Foundation Scientific Statement from the American Heart Association Council for High Blood Pressure Research Professional Education Committee, Council on Clinical Cardiology, Stroke Council, and Council On Cardiovascular Nursing. In collaboration with the National Heart, Lung, and Blood Institute National Center on Sleep Disorders Research (National Institutes of Health). Circulation. 2008 Sep 2;118(10):1080–111.

58. Peppard PE, Young T, Barnet JH, Palta M, Hagen EW, Hla KM. Increased prevalence of sleep-disordered breathing in adults. Am J Epidemiol. 2013 May 1;177(9):1006–14.

59. Gami AS, Pressman G, Caples SM, Kanagala R, Gard JJ, Davison DE, et al. Association of atrial fibrillation and obstructive sleep apnea. Circulation. 2004 Jul 27;110(4):364–7.

60. Stevenson IH, Teichtahl H, Cunnington D, Ciavarella S, Gordon I, Kalman JM. Prevalence of sleep disordered breathing in paroxysmal and persistent atrial fibrillation patients with normal left ventricular function. Eur Heart J. 2008 Jul;29(13):1662–9.

61. Fein AS, Shvilkin A, Shah D, Haffajee CI, Das S, Kumar K, et al. Treatment of obstructive sleep apnea reduces the risk of atrial fibrillation recurrence after catheter ablation. J Am Coll Cardiol. 2013 Jul 23;62(4):300–5.

62. ACPICR. Standards for physical activity and exercise in the cardiac population 2015. Available at: http://acpicr.com

63. Kato M, Ogano M, Mori Y, Kochi K, Morimoto D, Kito K, et al. Exercise-based cardiac rehabilitation for patients with catheter ablation for persistent atrial fibrillation: A randomized controlled clinical trial. Eur J Prev Cardiol. 2019 Jul 5;2047487319859974.

64. Fiala M, Bulková V, Šknouril L, Nevralová R, Toman O, Januška J, et al. Functional improvement after successful catheter ablation for long-standing persistent atrial fibrillation. Eur Eur Pacing Arrhythm Card Electrophysiol J Work Groups Card Pacing Arrhythm Card Cell Electrophysiol Eur Soc Cardiol. 2017 Nov 1;19(11):1781–9.

65. ESC/ESH Arterial Hypertension (Management of) Guidelines [Internet]. [cited 2020 Jan 31]. Available from: https://www.escardio.org/Guidelines/Clinical-Practice-Guidelines/Arterial-Hypertension-Management-of, https://www.escardio.org/Guidelines/Clinical-Practice-Guidelines/Arterial-Hypertension-Management-of.

66. J. Brazier, J. Roberts, M. Deverill. The estimation of a preference-based measure of health from the SF-36. J Health Econ, 21 (2002), pp. 271-292, 10.1016/S0167-6296(01)00130-8.

67. Barber JA, Thompson SG. Analysis of cost data in randomized trials: an application of the non-parametric bootstrap. Stat.Med. 2000; 19(23):3219-36.

68. Andronis L, Barton P, Bryan S. Sensitivity analysis in economic evaluation: an audit of NICE current practice and a review of its use and value in decision-making. Health Technol Assess. 2009; 13(29):iii, ix-xi, 1-61.

69. Glick HA, Jalpa DA, Sonnad SS, Polsky D. Economic evaluation in clinical trials. Oxford: Oxford University Press 2007.

70. Barton GR, Briggs AH, Fenwick EA. Optimal cost-effectiveness decisions: the role of the cost-effectiveness acceptability curve (CEAC), the cost-effectiveness acceptability frontier (CEAF), and the expected value of perfection information (EVPI). Value Health. 2008; 11(5):886-97.
