# Supplementary material for: Cardiac prehabilitation, rehabilitation and education in first-time atrial fibrillation (AF) ablation (CREED AF): Study protocol for a randomised controlled trial
Source: PLoS One. 2024 Oct 3;19(10):e0310951. doi: 10.1371/journal.pone.0310951 (PMC11449326; doi:10.1371/journal.pone.0310951)
Supplement: Attachment — Submitted filename: Response to Reviewers Plos One CREED AF Aug 2024.doc [file pone.0310951.s003.doc]

17^th^ August 2024

Luigi Sciarra

Academic Editor

PLOS ONE

**Re: PONE-D-24-22167**

**Cardiac prehabilitation, rehabilitation and education in first-time atrial fibrillation (AF) ablation (CREED AF): study protocol for a randomised controlled trial**

**PLOS ONE**

Dear Luigi

Many thanks for your reply. We would like to thank you and the reviewers for your comments. We have addressed each of the points raised as highlighted in red below and would like to resubmit our revised manuscript.

**Professor Faizel Osman MD FRCP FESC**

**(corresponding author on behalf of all co-authors)**

Consultant Cardiologist / Electrophysiologist

Honorary Professor of cardiology (Warwick Medical School)

Institute of Cardiometabolic Medicine

University Hospital Coventry

Clifford Bridge Road

Coventry

CV2 2DX UK

Tel: +44 2476 9645668

Email: Faizel.osman@uhcw.nhs.uk

**ACADEMIC EDITOR:**

Please submit your revised manuscript by Sep 13 2024 11:59PM. If you will need more time than this to complete your revisions, please reply to this message or contact the journal office at plosone@plos.org. When you're ready to submit your revision, log on to https://www.editorialmanager.com/pone/ and select the 'Submissions Needing Revision' folder to locate your manuscript file.

Please include the following items when submitting your revised manuscript:

•	A rebuttal letter that responds to each point raised by the academic editor and reviewer(s). You should upload this letter as a separate file labeled 'Response to Reviewers'. completed

•	A marked-up copy of your manuscript that highlights changes made to the original version. You should upload this as a separate file labeled 'Revised Manuscript with Track Changes'. completed

•	An unmarked version of your revised paper without tracked changes. You should upload this as a separate file labeled 'Manuscript'. completed

If you would like to make changes to your financial disclosure, please include your updated statement in your cover letter. Guidelines for resubmitting your figure files are available below the reviewer comments at the end of this letter.

If applicable, we recommend that you deposit your laboratory protocols in protocols.io to enhance the reproducibility of your results. Protocols.io assigns your protocol its own identifier (DOI) so that it can be cited independently in the future. We can perform this.

For instructions see: https://journals.plos.org/plosone/s/submission-guidelines#loc-laboratory-protocols. Additionally, PLOS ONE offers an option for publishing peer-reviewed Lab Protocol articles, which describe protocols hosted on protocols.io. Read more information on sharing protocols at https://plos.org/protocols?utm_medium=editorial-email&utm_source=authorletters&utm_campaign=protocols.

We look forward to receiving your revised manuscript.

Kind regards,

Luigi Sciarra

Academic Editor

PLOS ONE

**Journal requirements:**

When submitting your revision, we need you to address these additional requirements.

1. Please ensure that your manuscript meets PLOS ONE's style requirements, including those for file naming. The PLOS ONE style templates can be found at Completed

https://journals.plos.org/plosone/s/file?id=wjVg/PLOSOne_formatting_sample_main_body.pdf and https://journals.plos.org/plosone/s/file?id=ba62/PLOSOne_formatting_sample_title_authors_affiliations.pdf

Completed

2. We noticed you have some minor occurrence of overlapping text with the following previous publication(s), which needs to be addressed:

<https://www.ncbi.nlm.nih.gov/pmc/articles/PMC6020195/?report=abstract&tool=pmcentrez>

We have now cited this paper in our manuscript (page 5)

<https://www.birmingham.ac.uk/Documents/college-mds/trials/bctu/A-Renovascular/Basil-3-Protocol-v4.0-06-AUG-2019.pdf>

We have now referenced this protocol in our manuscript (page 15)

In your revision ensure you cite all your sources (including your own works), and quote or rephrase any duplicated text outside the methods section. Further consideration is dependent on these concerns being addressed.

3. We note that the grant information you provided in the ‘Funding Information’ and ‘Financial Disclosure’ sections do not match. We have declared funding received from Boston Scientific for this study in our manuscript (page 18) and in the Funding Information section during online submission.

When you resubmit, please ensure that you provide the correct grant numbers for the awards you received for your study in the ‘Funding Information’ section. No grant number has been provided to us by the funder

4. In the online submission form, you indicated that [No datasets were generated or analysed during the current study. All relevant data from this study will be made available upon study completion.].

All PLOS journals now require all data underlying the findings described in their manuscript to be freely available to other researchers, either 1. In a public repository, 2. Within the manuscript itself, or 3. Uploaded as supplementary information.

This policy applies to all data except where public deposition would breach compliance with the protocol approved by your research ethics board. If your data cannot be made publicly available for ethical or legal reasons (e.g., public availability would compromise patient privacy), please explain your reasons on resubmission and your exemption request will be escalated for approval. We confirm all data will be freely available

5. Please review your reference list to ensure that it is complete and correct. If you have cited papers that have been retracted, please include the rationale for doing so in the manuscript text, or remove these references and replace them with relevant current references. Any changes to the reference list should be mentioned in the rebuttal letter that accompanies your revised manuscript. If you need to cite a retracted article, indicate the article’s retracted status in the References list and also include a citation and full reference for the retraction notice. This has now been checked and completed

**Reviewer #1: PONE-D-24-22167: statistical review**

This is a study protocol for a randomised controlled trial to assess clinical-efficacy/cost-effectiveness of prehabilitation/rehabilitation/patient-education among subjects undergoing first time atrial fibrillation ablation. The protocol relies on clearly identified research questions that can be tested by the suggested primary and secondary outcomes. The methods are standard and reproducible, sample size calculations rely on clearly stated hyptheses and methods for possible missing values are declared. Overall, I believe that this is a well-designed study protocol. Thank you

**Reviewer #2:** Authors presented an ambitious project to evaluate whether supervised sports practice, before and after an atrial fibrillation ablation, can determine differences in terms of outcome.

Study design seem to be appropriate for the purpose. Thank you

A question can arise: have authors considered that some athletes, suffering from AF, can be enrolled in the study? Can this occurrence introduce a bias in results interpretation?

Please specify this issue.

The question about athletes, particularly high-endurance level athletes, did come to mind when planning and developing the research protocol for CREED AF. The general consensus was to maintain generalizability of the study by being inclusive to as many individuals suffering with atrial fibrillation (AF) undergoing first time AF ablation as possible.

Concern may rest with higher than average VO2 peak seen in these individuals when matched with non-athletes. It has been observed that high-endurance athletes do also suffer with AF, with reduced physical ability/fitness prior to ablation, with improvement of most to near previous levels of fitness/exertion after ablation (Liu et al., 2022). The application of the CREED AF intervention, which aims at delivering a moderate level of exercise (tailored to the individual) alongside risk factor management, is equally applicable in these potential participants. Furthermore, the primary outcome measure is mean change in VO2 peak from baseline to primary follow-up at 10 +/-2 weeks post-ablation in the two groups; measuring and comparing the change would minimize any bias, if any, derived by inclusion of high-endurance athletes.

We have now included this in the discussion section on page 17
